# Novel Insights Into Bacterial Dimethylsulfoniopropionate Catabolism in the East China Sea

**DOI:** 10.3389/fmicb.2018.03206

**Published:** 2018-12-21

**Authors:** Jingli Liu, Ji Liu, Sheng-Hui Zhang, Jinchang Liang, Heyu Lin, Delei Song, Gui-Peng Yang, Jonathan D. Todd, Xiao-Hua Zhang

**Affiliations:** ^1^College of Marine Life Sciences, Ocean University of China, Qingdao, China; ^2^School of Biological Sciences, University of East Anglia, Norwich, United Kingdom; ^3^College of Chemistry and Chemical Engineering, Ocean University of China, Qingdao, China; ^4^Laboratory for Marine Ecology and Environmental Science, Qingdao National Laboratory for Marine Science and Technology, Qingdao, China

**Keywords:** DMSP catabolism, DMS, methanthiol (MeSH), bacterial community, the East China Sea

## Abstract

The compatible solute dimethylsulfoniopropionate (DMSP), made by many marine organisms, is one of Earth's most abundant organosulfur molecules. Many marine bacteria import DMSP and can degrade it as a source of carbon and/or sulfur via DMSP cleavage or DMSP demethylation pathways, which can generate the climate active gases dimethyl sulfide (DMS) or methanthiol (MeSH), respectively. Here we used culture-dependent and -independent methods to study bacteria catabolizing DMSP in the East China Sea (ECS). Of bacterial isolates, 42.11% showed DMSP-dependent DMS (Ddd^+^) activity, and 12.28% produced detectable levels of MeSH. Interestingly, although most Ddd^+^ isolates were *Alphaproteobacteria* (mainly Roseobacters), many gram-positive *Actinobacteria* were also shown to cleave DMSP producing DMS. The mechanism by which these *Actinobacteria* cleave DMSP is unknown, since no known functional *ddd* genes have been identified in genome sequences of Ddd^+^ *Microbacterium* and *Agrococcus* isolates or in any other sequenced *Actinobacteria* genomes. Gene probes to the DMSP demethylation gene *dmdA* and the DMSP lyase gene *dddP* demonstrated that these DMSP-degrading genes are abundant and widely distributed in ECS seawaters. *dmdA* was present in relatively high proportions in both surface (19.53% ± 6.70%) and bottom seawater bacteria (16.00% ± 8.73%). In contrast, *dddP* abundance positively correlated with chlorophyll *a*, and gradually decreased with the distance from land, which implies that the bacterial DMSP lyase gene *dddP* might be from bacterial groups that closely associate with phytoplankton. Bacterial community analysis showed positive correlations between *Rhodobacteraceae* abundance and concentrations of DMS and DMSP, further confirming the link between this abundant bacterial class and the environmental DMSP cycling.

## Introduction

The tertiary sulfonium compound dimethylsulfoniopropionate (DMSP) is made in prodigious amounts (several petagrams, worldwide annually) in marine environments (Ksionzek et al., 2016). DMSP is synthesized by many marine microalgae, e.g., coccolithophores, dinoflagellates, and diatoms (Curson et al., 2018; Kageyama et al., 2018), macroalgae (Reed, 1983), a few angiosperms (Otte et al., 2004), and some corals (Raina et al., 2013). Recently, heterotrophic bacteria have also been reported to synthesize DMSP (Curson et al., 2017). In these organisms DMSP may function in e.g., storage of excess sulfur and carbon (Stefels, 2000), cryoprotection, oxidative damage protection (Sunda et al., 2002), signaling pathways acting as chemoattractant (Seymour et al., 2010) and enhancing the production of quorum-sensing molecules (Johnson et al., 2016). The major ecological significance of DMSP lies in it being an important nutrient for marine microorganisms (Curson et al., 2011b) providing carbon, sulfur and/or energy to microbes catabolizing it. Microbial DMSP catabolism can generate environmentally important catabolites including the climate active gases dimethylsulfide (DMS) and methanethiol (MeSH). DMS, largely derived from DMSP catabolism, is the most significant biogenic sulfur compound transferred from oceans to the atmosphere [~3 × 10^8^ tons, worldwide annually; (Andreae, 1990)], where its oxidative products act as cloud condensation nuclei (CCN) affecting cloud cover and thus the radiation reaching Earth's surface (Vallina and Simó, 2007).

Although many marine phytoplankton can catabolize DMSP (Alcolombri et al., 2015; Johnston, 2015), it is believed that marine bacteria are significant contributors to global DMSP catabolism once this molecule is released into the dissolved pool of DMSP in seawater (Curson et al., 2011b). Indeed, DMSP supports 1–13% of the bacterial carbon demand in surface seawater (Kiene and Linn, 2000) and it is especially important as a reduced organic sulfur source, e.g., for the dominant heterotrophic bacteria SAR11, which require exogenous sources of reduced sulfur for growth (Tripp et al., 2008). A wide variety of marine microorganism import (Vila et al., 2004; Howard et al., 2008) and catabolize DMSP via two enzymatic pathways: demethylation and cleavage (Curson et al., 2011b; Moran et al., 2012). Demethylation is believed to be the predominant DMSP catabolic pathway, converting ~75% dissolved DMSP into 3-methylmercaptopropionate (MMPA), further into MeSH, and then into microbial biomass (Kiene and Linn, 2000). This pathway does not liberate DMS. The maker gene for DMSP demethylation “*dmdA*” is only found in bacteria and is prevalent in the SAR11 lineage and another abundant marine *Alphaproteobacteria* lineage known as the Roseobacters (Howard et al., 2006; Reisch et al., 2011). The *dmdA* genes can be grouped into five clades and fourteen subclades based on their nucleotide and amino acid sequences (Howard et al., 2006, 2008; Varaljay et al., 2010).

By comparison, there is far more biodiversity in the DMSP cleavage pathway where DMSP lyase enzymes generate DMS from DMSP in bacteria, some fungi and phytoplankton (Curson et al., 2011b; Alcolombri et al., 2015; Sun et al., 2016). To date, eight different DMSP lyase genes, *dddD, dddL, dddP, dddQ, dddW, dddY, dddK*, and *Alma1*, encoding distinct polypeptides in various protein families, have been identified in a wide range of microbes, demonstrating a high level of biochemical and genetic diversity in DMSP lyase enzymes (Curson et al., 2011b; Alcolombri et al., 2015; Johnston, 2015; Johnston et al., 2016; Sun et al., 2016). With the exception of *dddY*, the bacterial *ddd* genes are common in Roseobacters (Curson et al., 2011b), which can account for up to 30% of bacterioplankton cells (Gonzalez and Moran, 1997) in eutrophic coastal regions where DMS emission is intense and likely exerts influence on climate, e.g., the East China Sea (ECS). Of the identified bacterial DMSP lyases, the DMSP lyase genes *dddP* and *dddQ* are by far the most prevalent in ocean microbial reference gene catalog (OM-RGC) metagenomic and Tara Oceans metatranscriptomic datasets apportioned mainly to marine bacteria (Curson et al., 2018).

**Figure 1:**
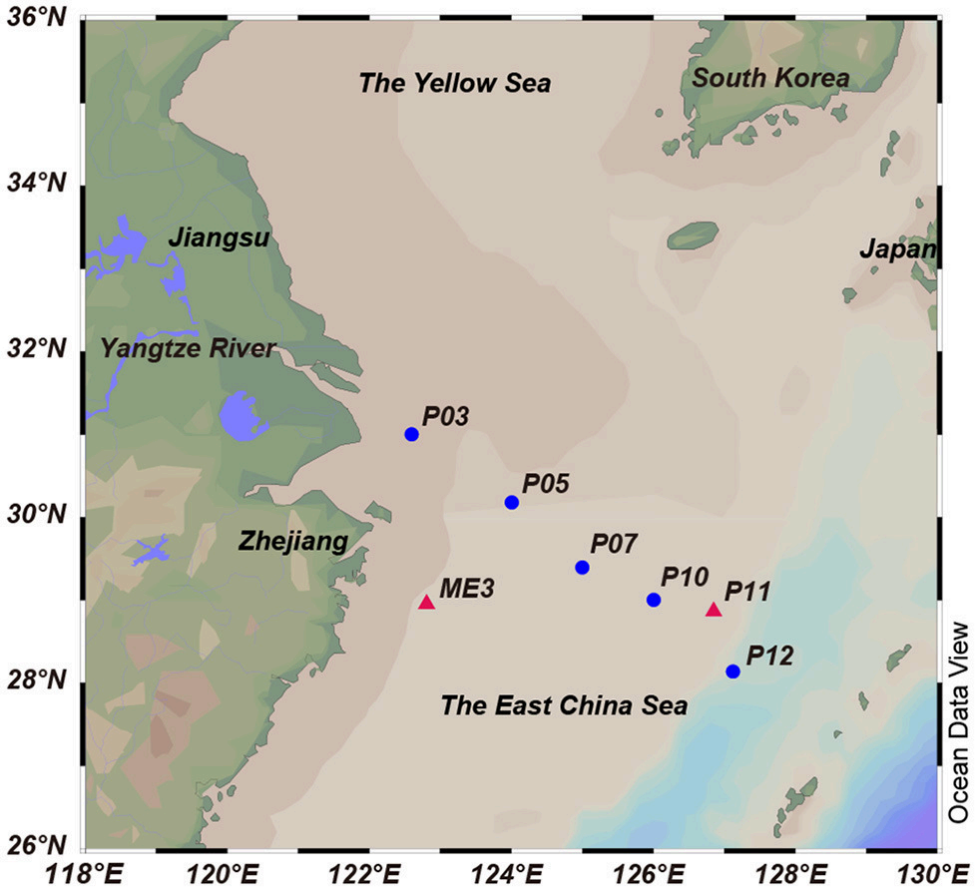
Locations of the sampling sites in the ECS. The red triangles indicate sites from 2013 cruise, and the blue circles indicate sites from 2015 cruise. Stations plotted in Ocean Data View (Schlitzer, 2002).

The ECS is the largest marginal sea of the western Pacific (Figure 1). It is influenced by the Yangtze River effluent and the Kuroshio water current, and acts as a transition zone where terrigenous and anthropogenic materials are discharged from the mainland to the ocean. A previous study of the ECS in the summer of 2011 found the surface waters to contain moderate concentrations of DMSP, DMS and chlorophyll *a* (28.25 nM, 5.64 nM and 0.84 mg L^−1^, respectively) (Yang et al., 2011). To date, our understanding of microbial DMSP metabolism in marine environments comes mainly from studies conducted in open sea, salt marsh and estuarine environments (e.g., Ansede et al., 2001) or with phytoplankton-attached bacteria (e.g., Hatton et al., 2012). Studies on the spatial and temporal distribution of bacterial DMSP-degrading genes have been carried out in the Sargasso Sea (Levine et al., 2012), the Pacific Ocean (Varaljay et al., 2012; Cui et al., 2015), and the Arctic Kongsfjorden (Zeng et al., 2016). However, studies of the abundance and diversity of bacterial DMSP catabolism in marginal sea environments are limited. In this study, we collected and characterized seawater samples from seven ECS sites for their oceanographic parameters and DMSP compositions. Culture-dependent methods were used to study DMSP catabolizing bacteria in samples from two of these sites and led to the identification of novel DMSP catabolizing bacterial taxa. Culture-independent methods were used to explore the spatial distribution and diversity of key DMSP catabolic genes in a transect of five ECS stations from inshore to the offshore waters, and the results further demonstrate the importance of bacterial DMSP-catabolism in the ECS.

## Materials and Methods

### Sampling and Environmental Parameters

Surface seawater (SW) and bottom seawater (BW) samples were collected onboard the R/V “*Dong Fang Hong 2”* in the ECS during two cruises from 14 July to 1 August 2013 (two sites, ME3 located near the continent and P11 further from the land) and 19 October to 2 November 2015 (five sites, P03, P05, P07, P10, and P12; along a transect from coast to the ocean), respectively (Figure 1, Table 1). Seawater was collected by Niskin bottles equipped on a standard conductivity-temperature-depth rosette (CTD). One liter of each sample was immediately filtered through 0.22 μm pore size polycarbonate membranes filters (Millipore Corporation, Billerica, MA, USA). Filters from 2013 cruise were soaked in sterile 0.85% (w/v) saline supplemented with 15% (v/v) glycerol before stored at −80°C until in-lab bacterial isolation. Filters from 2015 cruise were stored in liquid nitrogen onboard and at −80°C in lab for nucleic acid extraction. Salinity, temperature and dissolved oxygen were recorded with a Seabird 911 conductivity-temperature-depth (CTD).

**Table 1:** Seawater sampling stations and environmental parameters.

**Station**	**Latitude (^**°**^N)**	**Longitude (^**°**^E)**	**Sampling year**	**Sampling layer*^***a***^***	**Depth (m)**	**T (^**°**^C)**	**Salinity (ppt)**	**DO (mg/L)**	**Chl *a* (μg/L)**	**DMSPt (nM)**	**DMSPd (nM)**	**DMSPp (nM)**	**DMS (nM)**
P03	31.00166	122.5998	2015	SW	4	22.05	30.13	6.9955	0.69	88.42	4.63	83.78	5.36
				BW	50	23.46	33.73	4.7251	0.57	8.63	2.68	5.95	1.62
P05	30.17909	124.0094	2015	SW	3	24.07	32.59	5.149	0.54	15.59	1.8	13.79	2.17
				BW	45	24.07	33.75	6.2535	0.36	8.1	3.08	5.02	1.35
P07	29.39963	125.0007	2015	SW	5	24.35	33.77	6.4402	0.29	NA	NA	NA	NA
				BW	76	22.32	34.24	3.8145	0.16	NA	NA	NA	NA
P10	29.0014	126.0038	2015	SW	3	24.54	33.76	6.4949	0.36	24.03	2.77	21.26	2.2
				BW	94	19.6	34.62	5.0794	0.04	8.1	3.62	4.48	1.34
P12	28.13561	127.1214	2015	SW	4	26.44	34.85	6.3014	0.14	10.4	2.31	8.09	0.96
				100 m	100	23.57	34.91	6.3717	0.21	NA	NA	NA	NA
				BW	999	4.341	34.41	2.9671	NA	NA	NA	NA	NA
ME3	28.97528	122.8178	2013	SW	3	26.56	33.63	6.6998	5.28	38.36	6.67	31.69	5.54
				BW	59	18.36	34.41	5.0263	0.22	11.45	3.34	8.11	2.12
P11	28.87861	126.8517	2013	SW	3	28.9	33.61	6.0879	0.2	10.23	2.56	7.67	2.11
				BW	193	11.83	34.39	4.7463	0.03	3.22	0.99	2.23	0.89

a *“SW” stands for surface seawater; “BW” stands for bottom seawater, “NA” stands for not analyzed*.

DMS and DMSP concentrations in the seawater samples were measured as described by Zhang et al. (2014). DMS samples were measured onboard immediately after sampling using a modified purge and trap method. Briefly, a sample of 2 mL was collected into a glass bubbling chamber through a GF/F filter. Sulfur gases were sparged from the seawater with nitrogen and trapped in a loop of Teflon tubing immersed in liquid nitrogen. The trapped gases were desorbed with hot water (90°C) and analyzed on a Shimadzu GC-2014 gas chromatograph equipped with a flame photometric detector. A 3 m × 3 mm glass column packed with 10% DEGS on Chromosorb W-AW-DMCS was used to separate sulfur gases at 70°C.

Gravity filtering of samples for dissolved DMSP (DMSPd) was conducted as described by Kiene and Slezak (2006) with the following modifications. Total DMSP (DMSPt) and DMSPd samples were fixed with 50% sulfuric acid and stored on ship at room temperature for 2 days. For DMSPd samples, the first few drops of filtrate was discarded, and a 4 mL sample was transferred to a glass vial containing 40 μL of concentrated sulfuric acid and sealed. For DMSPt samples, 100 μL of 50% sulfuric acid was directly added to 10 mL of unfiltered seawater samples and then sealed. When analyzed, 300 μL of 10 M KOH was injected into 2 mL of the preserved DMSP sample and incubated in the dark at 4°C for at least 24 h, allowing for complete conversion of DMSP into DMS and acrylate. The liberated DMS was measured using the method described above.

For chlorophyll a (Chl *a*) analysis, the seawater samples were filtered through 47 mm Whatman GF/F filters. The filters were soaked in 10 ml of 90% acetone and then stored in the dark at 4°C. After 24 h, the concentration of Chl *a* was measured using a F4500 (Hitachi) fluorometer (Parsons et al., 1984).

### Bacteria Isolation and Phylogenetic Analysis

Filters from the 2013 cruise were rinsed with sterile 0.85% (w/v) saline supplemented with 15% (v/v) glycerol. The cells were resuspended by vortexing, spread on Marine Agar (MA) plates and incubated at 28°C for 1 week. Single colonies were picked randomly and purified three times on fresh plates prior to further studies. Genomic DNA of the isolates was extracted by phenol/chloroform extraction, and the 16S rRNA genes were amplified using the 27F/1492R primer set (Lane, 1991) and sequenced to determine their taxonomy. Calculation of pairwise similarity values for the 16S rRNA gene of the cultivated strains and the most closely related type strains were achieved from the Ezbiocloud server (http://www.ezbiocloud.net/identify). The 16S rRNA gene sequences of representative cultivated bacterial strains and the most closely related type strains were aligned using the CLUSTAL_X program (Thompson et al., 1997). Phylogenetic trees based on the neighbor-joining (NJ) algorithms were constructed by MEGA version 5.0 (Tamura et al., 2011) with K2+G model. The tree topology was checked by 1000 bootstrap replicates.

### Sole Carbon Source Test

Fifty-seven representative bacterial isolates were selected and tested for their growth on MBM minimal medium (Baumann and Baumann, 1981) with DMSP (2 mM; TCI, Japan) as sole carbon source. The same medium lacking a carbon source (negative control) or supplied with glucose (2 mM), succinate (2 mM), sucrose (2 mM), pyruvic acid sodium salt (2 mM), and glycerol (2 mM) as the carbon source was used. Briefly, cells were grown in Marine Broth (MB; Becton Dickinson) and harvested after incubation at 28°C for 2 days, and then washed three times with 3% (w/v) NaCl saline. Washed cells were diluted to OD_600nm_ = 0.4–0.6, then 1% (w/v) were inoculated in triplicate into the media supplied with DMSP or mixed carbon source. Cells added to MBM medium without any carbon source were set up as non-carbon control. Growth was measured spectrophotometrically (OD_600nm_) after 1 week. Significance was determined using a Student's *t*-test (*P* < 0.05).

### DMSP Catabolism Assay

To measure DMSP catabolism of cultivated bacteria, bacterial strains were grown overnight in MB at 28°C. Cells were washed twice in MAMS media (Table S1) and diluted to OD_600_ = 0.3 prior to 1 in 10 dilution into vials (CNW, China) containing MAMS media supplied with 0.45% glycerol (v/v) and 0.05% glucose (w/v) as the mixed carbon source and 1 μM, 0.5 mM or 5 mM DMSP as the catabolism substrate to get a final volume of 2 mL. After gas-tight sealing and incubation at 28°C for ~36 h, the 2 mL cultures were directly assayed for DMS and MeSH production as described above. Ratified DMSP degrading strains, *Roseovarius nubinhibens* ISM and *Ruegeria pomeroyi* DSS-3, which can produce both DMS and MeSH from DMSP (González et al., 1999, 2003), were used as positive controls. Abiotic media controls of MAMS media supplied with the same concentration of DMSP were set up and incubated under the same conditions to monitor the background chemical lysis of DMSP to DMS. Bacterial Ddd production was calculated by subtracting the abiotically generated DMS from the total detected DMSP-dependent DMS in the bacterial cultures.

An eight-point (2.06–103 nM) calibration curve was made with a gradient DMS concentrations to calculate the DMSP dependent DMS production rate of tested bacterial strains. The detection limit for the purge and trap GC analysis of DMS was 0.8 pmol DMS, and the square root of the DMS peak area was linear to the DMS concentration. Total protein concentration in the cells was estimated using Bradford assays (Bio-Rad). The rate of DMS production was expressed in nmol DMS per mg protein per hour.

A similar eight-point (10–0.1 mM) calibration curve was constructed for MeSH as was done for DMS, see above. However, unlike DMS, the detected peak area of MeSH was not linear with MeSH concentration. The detection limit for the purge and trap GC analysis of MeSH was 0.1 μmol. Thus, MeSH assays carried out here, as described above, are considered as only qualitative and not quantitative for DMSP dependent MeSH production.

Strains that could use DMSP as sole carbon source were tested for their DMSP dependent DMS and MeSH production with and without the addition of mixed carbon source to MBM media (as above) to test whether the addition of extra carbon source significantly affected MeSH and/or DMS production from DMSP.

### BLASTp Analysis of Ddd and DmdA Homologs

The bacterial genome sequences of the strains that are of the same genus as our 57 representative isolates were retrieved from the NCBI database as reference genomes (https://www.ncbi.nlm.nih.gov/assembly, Table S2). Ratified DMSP lyases, i.e., DddD, DddL, DddP, DddQ, DddY and DddW, and demethylase DmdA (Table S3) were used to interrogate the putative proteins in all reference genome sequences, using the BLASTp program (E-value ≤ 1e-5), and then the produced sequences were manually curated with the thresholds of identity ≥ 40%, coverage ≥ 70%, and length difference ≤ 20%.

### Degenerate *dddP* Primer Design, PCR Amplification and Sequencing of *ddd* and *dmdA* genes of DMSP-Degrading Isolates

DMSP lyase coding genes *dddD, dddL* and *dmdA* were PCR amplified using the published primer pairs *dddD*f/*dddD*r, *dddL*f/*dddL*r (Raina et al., 2009) and *dmdA* primers for subclade A/1, A/2, B/3, and E/2 which contain culturable bacterial *dmdA* sequences (Varaljay et al., 2010), while the degenerate primer set DddPUf (ATGTTCGACCCGATGAACathmgntaygc) and DddPUr (CCGCACTCCTGGAACcanggrttngt) (Table S4) for *dddP* were acquired by the j-CODEHOPE designer (Rose et al., 2003; Boyce et al., 2009) based on the ratified DddP sequences in Table S3 and the validity of their *dddP*-targeting property was verified on *Ruegeria pomeroyi* DSS-3 and *Roseovarius nubinhibens* ISM as positive controls and sequenced marine strains without *dddP* in their genomes as negative controls. The PCR system included 250 μM of each deoxyribonucleotide triphosphate and 5 μL of 10 × rTaq buffer containing MgCl_2_, 0.4 μM of each primer, 1 U of rTaq DNA polymerase of TaKaRa (5 U/μL), ~50 ng bacterial genomic DNA and nuclease-free water to adjust to a final volume of 50 μL. The reaction conditions for *dddD* and *dddL* were the same as used by Raina et al. (2009) except that two rounds of PCR (using the product of the first round as template for the second round) were performed to enhance the intensity of the product. For *dddP*, two rounds of PCR were also performed and the reaction condition for each round was as follows: 95°C for 5 min; followed by 30 cycles of 95°C for 1 min, 58°C for 1 min, and 72°C for 40 s; and then a final extension of 72°C for 10 min. PCR products were visualized by electrophoresis on 1% agarose gel, purified by TIANgel Mini Purification Kit (TIANGEN Biotech, Beijing), then cloned into the pUCm-T (TaKaRa) and sequenced by M13F primer (Table S4) using an automated DNA sequencer (model ABI3730; Applied BioSystems) at BGI, Qingdao, China. All PCR amplicons were subjected to BLASTx analysis against the RefSeq database and hits were counted as encoding functional Ddd or DmdA sequence if they were most similar, ≥68% amino acid identity, to ratified Ddd or DmdA enzymes.

### Genomic Sequencing of Two Representative Actinobacteria

Genomic DNA of *Microbacterium* sp. ZYF042 and *Agrococcus* sp. LZB059 were extracted using E.Z.N.A. Bacterial DNA kit (Omega). Genome sequencing was performed by Shanghai Majorbio Bio-Pharm Technology Co. (China) using the Illumina HiSeq 2000 sequencer system with a 500 bp pair-end library. The reads were assembled using SOAPdenovo v2.04. The genome coverages for strains ZYF042 and LZB059 were 247 × and 450 ×, respectively. Putative genes were identified using Glimmer 3.02. Annotation was performed with BLAST+2.2.24, searching against the National Center for Biotechnology Information (NCBI) Non-Redundant Proteins (NR), Clusters of Orthologous Groups of Proteins (COG), the Kyoto Encyclopedia of Genes and Genomes (KEGG) and Gene Ontology (GO) databases, respectively. Ddd and DmdA homolog searching was performed by BLASTP analysis with verified Ddd sequences as query sequences, as above (Table S3).

### Cloning and Expression of Predicted *ddd*-Like Genes

The predicted *dddL* gene of *Ahrensia* LZD062 and *dddD*-like gene of *Microbacterium* ZYFD042 were amplified from their genomic DNA and ligated into the pET24a (+) vector (Novagen), transformed into *E.coli* BL21 (DE3) and incubated at 37°C in Luria-Bertani (LB) complete medium (Sambrook et al., 1989) supplemented with 100 μg mL^−1^ kanamycin. At the mid-exponential growth phase, isopropyl β-D-1-thiogalactopyranoside (IPTG) was added at a final concentration of 0.1 mM. Cultivation was continued at 16°C and 150 rpm until the cell density reached an OD_600_ of 1.2. To measure Ddd^+^ of recombinant *E. coli* cells, IPTG-induced culture was washed twice by M9 medium (Sambrook et al., 1989), then resuspended in M9 medium containing a final concentration of 500 nM DMSP and mixed carbon source as above, following by incubation with shaking in vials at 37°C. After 2 h, DMS was quantified by GC as described above.

### Environmental DNA Extraction, Pyrosequencing and Data Analysis

Total DNA of seawater samples were extracted using the method described by Yin et al. (2013) with a modified step to maximize the output, in which a Fast Prep-24 Homogenization System (MP Biomedicals, Irvine, CA, USA) was used to intensify cell lysis at maximum speed for 1 min. The V4-V5 regions of bacterial 16S rRNA gene were amplified with primer sets 515F/907R (Chen et al., 2016) in triplicate and pooled. Pyrosequencing was performed on Illumina MiSeq PE300 platform at Majorbio Bio-Pharm Technology Co., Ltd., Shanghai, China. Chimeras were excluded during assigning OTUs based on 97% similarity level. Taxonomic assignment was against the SILVA database (Release 123) with 80% similarity threshold. Archaeal 16S rRNA gene sequences were removed. After rarefaction to the minimum sequence number for each sample, estimators such as Chao 1′, Shannon indexes, and Good's coverage were calculated (Table S5). All the above analyzes were performed via Qiime pipelining (Caporaso et al., 2010).

### Quantification of *dddP, dmdA* and 16S rRNA Genes

qPCR was performed on StepOne ABI (Applied Biosystems, Foster City, CA, USA). The abundance of *dddP* was quantified with primer sets dddP_874F/dddP_971R (Table S4) targeting Roseobacter clade (Levine et al., 2012). Seven primer sets designed to target different *dmdA* subclades (A/1, A/2, B/3, D/1, D/3, C/2, E/2) were used for *dmdA* quantification (Varaljay et al., 2010). Environmental sample SW of P03 were subjected to Sanger sequencing to confirm *dmdA* and *dddP* gene specificity. The abundance of 16S rRNA gene was quantified using the primer set Eub338F/518R (Yin et al., 2013) (Table S4). All PCR reactions were performed in triplicates in 20 μL system using 10 μL 2 × SYBR Premix Ex Taq II (Takara Bio Inc.), 0.4 μL 50 × ROX reference dye, 0.2–0.4 μM each primer, 2 μL 1/10 diluted template DNA. The PCR reaction conditions referred to the primer designers. Amplification efficiencies for each gene ranged from 0.70 to 0.93, with all *R*^2^ values higher than 0.99. The relative abundance of bacterial DMSP-degrading genes was acquired by normalizing their copy numbers to the copy number of bacterial 16S rRNA gene. Pearson's correlation analysis was conducted among environmental parameters, DMSP-degrading gene relative abundance and 16S rRNA gene abundance in pyrosequencing data using R's Hmisc package. Statistical significance of the differences of functional gene relative abundance and 16S rRNA gene abundance between SW and BW samples were tested by Student *t*-test.

### Construction and Analyzes of *dddP* Clone Libraries

To study the diversity of *dddP, dddP* amplicons from different samples with primers designed by Peng et al. (2012) (Table S4) were used to construct clone libraries and the insertions in the vector were sequenced. The procedures were essentially as what described by Yin et al. (2013). The OTUs of *dddP* was determined with nucleotide similarity of 80% by Mothur. Estimators like Shannon, Simpson indexes and Good's coverage were calculated (Table S6). Representative sequences of each OTU were translated into protein sequences and used for phylogenetic tree construction as described above with the Poisson model.

## Data Availability

Bacterial 16S rRNA gene sequences and accompanying metadata produced from pyrosequencing were deposited in the NCBI Short Read Archive database under accession number SRP138803. Partial 16S rRNA genes of cultivated bacterial isolates were under the GenBank accession numbers KP639130 to KP639182; partial sequences of *dddP* gene from clone libraries were under accession numbers MH193618 to MH193931; amplified partial *dddL* genes from cultivated strains were under accession numbers MH193936 to MH193939, amplified *dddP* genes from cultivated strains were under accession numbers MH193932 to MH193935, amplified *dmdA* genes from cultivated isolates were under accession numbers MH193940 to MH193947. The Whole Genome Shotgun project of *Microbacterium* ZYFD042, *Agrococcus* sp. LZB059 have been deposited at DDBJ/ENA/GenBank under the accession RBZY00000000 and RBZZ00000000 respectively.

## Results

### Environmental Characteristics of the ECS Seawater

The oceanographic parameters, environmental factors and DMSP/DMS concentrations of the seawater samples were recorded (Table 1). As expected, SW samples had higher Chl *a*, DMSP, and DMS concentrations than BW samples. Coincidently, bacterial numbers, estimated by 16S rRNA gene quantitative PCR (qPCR), were also much higher in SW than in BW samples (3-fold higher in average) (Table S7, Figure S1). Chl *a* concentration ranged from 0.03 μg/L to 5.28 μg/L (average 0.65 μg/L) and showed a negative correlation with longitude/distance from shore (*r* = −0.78, *P* < 0.01) and salinity (*r* = −0.84, *P* < 0.05) (Figure S2). The average total DMSP (DMSPt) concentration in SW samples was 31.17 nM (ranging from 10.23 nM to 88.42 nM), which was ca. four times higher than that in BW samples (average 7.90 nM). Particulate DMSP (DMSPp) accounted for 55.31 to 94.75% (75.76% on average) of the DMSPt. DMS concentrations ranged from 0.89 to 5.54 nM (3.06 nM in SW and 1.46 nM in BW on average). These results are consistent with the higher light levels in SW over BW favoring photosynthetic algae which are thought to be the major producers of DMSP. This would in turn result in the higher levels of DMSP substrate observed in SW and ultimately higher microbial DMSP dependent DMS production.

### Isolation of Marine Bacteria From the ECS Seawater

**Figure 2:**
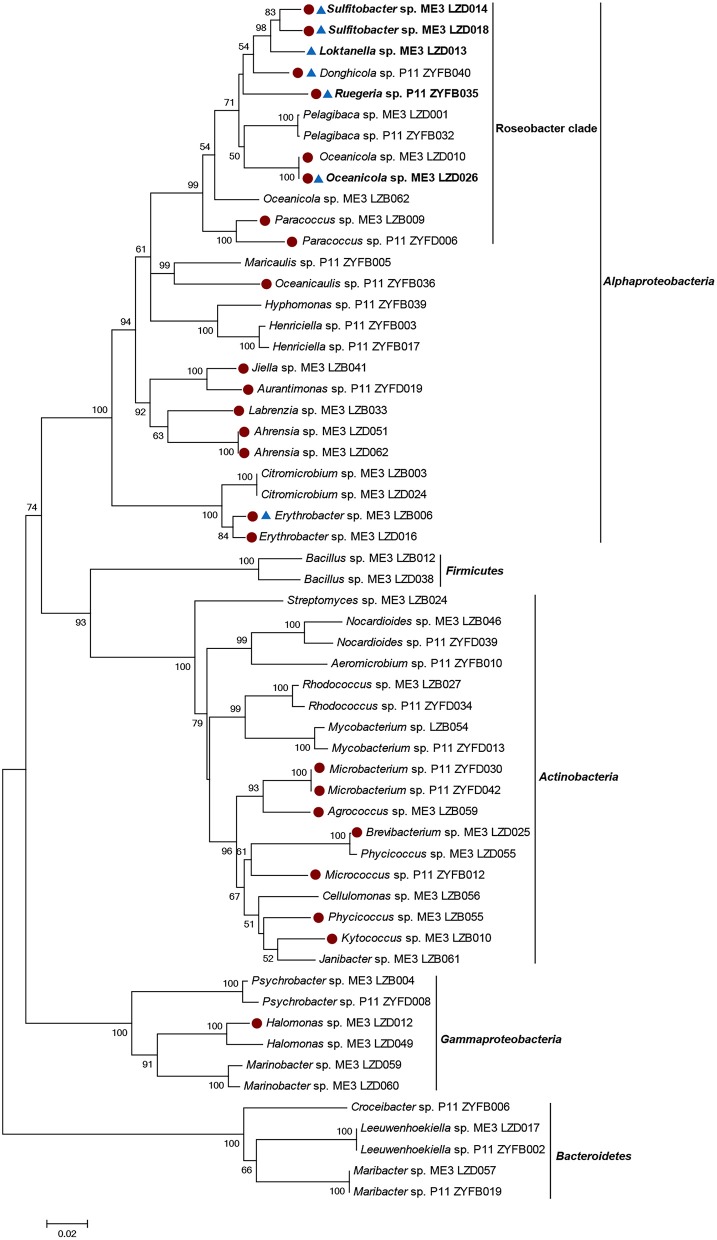
Neighbor-joining tree of 16S rRNA gene sequences of 57 representative cultivated strains. Solid circle represents Ddd^+^ strains. Triangle represents Ddm^+^ strains. The genera shown in bold represents bacteria which only showed Ddm^+^ activity when 0.5 and 5 mM DMSP was added. Bootstrap coefficients below 50% were not shown. Scale bar 0.02 substitutions per nucleotide position.

Heterotrophic marine bacteria from sites ME3 and P11 seawater were isolated on MA plates. There was no additional selective pressure applied during the incubation and isolation processes, enabling us to later assess the proportion of cultivable marine bacteria that could catabolize DMSP. In total 211 bacterial strains were isolated and identified from their 16S rRNA gene sequences. These isolates belonged to the phyla *Proteobacteria, Actinobacteria, Bacteroidetes*, or *Firmicutes*, comprising 37 genera and 54 species (Figure 2). *Alphaproteobacteria* comprised the largest percentage of bacterial isolates, accounting for 60.66% of the total cultivated bacterial community (73.60% in ME3 and 41.86% in P11). Not surprisingly, Roseobacters were well represented within the isolates, accounting for 29.86% (35.20% in ME3 and 22.09% in P11), while *Gammaproteobacteria* accounted for 5.2% of the total bacterial isolates (6.40% in ME3 and 3.49% in P11). In addition, *Actinobacteria* (22.33% in total, 12.80% in ME3 and 36.05% in P11), *Bacteroidetes* (9.95% in total, 4.00% in ME3 and 18.60% in P11), and *Firmicutes* (1.90% in total, 3.20% in ME3 and none in P11) were also represented. Very little is known about DMSP catabolism in any *Bacteroidetes, Actinobacteria* or *Firmicutes* and, to our knowledge, none of these bacteria are known to contain functional *dmdA* or any DMSP lyase genes.

### DMSP Catabolism by Cultivated Bacterial Strains and Their *ddd* and *dmdA* Genes

Fifty-seven representative isolates from the different genera were screened for their ability to utilize DMSP as sole carbon source (Figure 2; Table S2). Of these bacterial isolates, only alphaproteobacterial strains of the genera *Sulfitobacter* and *Ahrensia*, and gammaproteobacterial *Halomonas* could use DMSP as sole carbon source (Table S8). Some *Halomonas* isolates, e.g., HTNK1 are known to use DMSP as sole carbon source, cleave DMSP and contain the CoA transferase family DMSP lyase DddD (Todd et al., 2010). We also tested the ability of these strains to degrade DMSP with and without a mixed carbon source. The strains of genera *Ahrensia* and *Halomonas*, which only produced DMS from DMSP, had much higher DMS production when only supplied with DMSP as sole carbon source compared to the presence of the mixed carbon sources (Figure S3). The same was true for *Sulfitobacter* sp. LZD018, which produced comparatively higher levels of MeSH when DMSP was used as sole carbon source. Note the MeSH could not be accurately quantified by the GC method used here, likely due to the highly reactive properties of MeSH. These observations implied that the ability of these strains to catabolize DMSP is likely underestimated if catabolism is assayed in the presence of other carbon sources.

It is well known that many bacteria containing functional *ddd* and/or *dmdA* genes are not able to utilize DMSP as a sole carbon source under lab conditions (Curson et al., 2011b). For this reason, all the representative isolates were tested for their DMSP-dependent DMS (Ddd) and MeSH production (Ddm) when grown in the presence of mixed “regular” carbon sources (0.45% [v/v] glycerol and 0.05% [w/v] glucose). Under this condition, 24 (42.11%) of the tested isolates showed Ddd^+^ activity (Figure 2 Table 2; Table S2).

**Table 2:** Characteristics of DMSP-degrading bacterial isolates from the ECS sites ME3 and P11 and their proportions in the total bacteria community.

**Representative strain code**	**Closest taxonomically related genus**	**Strain source**	**Taxonomic description**	**Rate of DMSP-dependent DMS production^**a**^**	**Ddm^**+**^ activity^**b**^**	**PCR detected *ddd*/*dmdA***	**Known Ddd/DmdA homologs in genome sequenced strains in the same genera**	**Average proportions of the genus against total bacteria**
ZYFB040	*Donghicola*	P11 SW	*Alphaproteobacteria;* Roseobacter	32.37 ± 5.41	Y	*dddP*	DddW	0%
LZD013	*Loktanella*	ME3 BW	*Alphaproteobacteria;* Roseobacter	N.D.*^*c*^*	Y	*dmdA A/2*	DddD, DddL,DddW,DmdA	0%
LZD026	*Oceanicola*	ME3 BW	*Alphaproteobacteria;* Roseobacter	97.15 ± 0.19	Y	*dddL*	DddL, DmdA	0%
LZD010	*Oceanicola*	ME3 BW	*Alphaproteobacteria;* Roseobacter	97.22 ± 1.49	N.D.	*dddL*		
LZB009	*Paracoccus*	ME3 SW	*Alphaproteobacteria;* Roseobacter	8.43 ± 0.53	N.D.	N.D.	DddP	0.70 ± 0.59%
ZYFD006	*Paracoccus*	P11 BW	*Alphaproteobacteria;* Roseobacter	13.05 ± 2.84	N.D.	N.D.		
ZYFB035	*Ruegeria*	P11 SW	*Alphaproteobacteria;* Roseobacter	47.07 ± 7.07	Y	*dddP*	DddD, DddP, DddW, DmdA	0.84 ± 0.57%
LZD014	*Sulfitobacter*	ME3 BW	*Alphaproteobacteria;* Roseobacter	459.48 ± 209.96	Y	*dddL, dmdA A/2*	DddL, DmdA	0.70 ± 0.41%
LZD018	*Sulfitobacter*	ME3 BW	*Alphaproteobacteria;* Roseobacter	19.06 ± 7.01	Y	*dddP*		
LZD051	*Ahrensia*	ME3 BW	*Alphaproteobacteria*	6122.78 ± 297.96	N.D.	N.D.	DddP, DmdA	0%
LZD062	*Ahrensia*	ME3 BW	*Alphaproteobacteria*	532.83 ± 65.12	N.D.	N.D.		
ZYFD019	*Aurantimonas*	P11 BW	*Alphaproteobacteria*	25.67 ± 21.86	N.D.	N.D.	N.D.	0.04 ± 0.01%
LZB006	*Erythrobacter*	ME3 SW	*Alphaproteobacteria*	10.07 ± 6.46	Y	N.D.	N.D.	0.15 ± 0.22%
LZD016	*Erythrobacter*	ME3 BW	*Alphaproteobacteria*	35.36 ± 7.37	N.D.	N.D.		
LZB041	*Jiella*	ME3 SW	*Alphaproteobacteria*	28.13 ± 0.46	N.D.	N.D.	N.D.	0%
LZB033	*Labrenzia*	ME3 SW	*Alphaproteobacteria*	15.14 ± 0.32	N.D.	*dddL*	DddL, DddP, DmdA	0.05 ± 0.04%
ZYFB036	*Oceanicaulis*	P11 SW	*Alphaproteobacteria*	14.65 ± 8.62	N.D.	N.D.	N.D.	0%
LZD012	*Halomonas*	ME3 BW	*Gammaproteobacteria*	6.14 ± 1.29	N.D.	N.D.	DddP	1.5 ± 0.01%
LZB059	*Agrococcus*	ME3 SW	*Actinobacteria*	907.91 ± 339.92	N.D.	N.D.	N.D.	0%
LZD025	*Brevibacterium*	ME3 BW	*Actinobacteria*	18.99 ± 5.10	N.D.	N.D.	N.D.	0.02 ± 0.01%
LZB010	*Kytococcus*	ME3 SW	*Actinobacteria*	15.29 ± 2.21	N.D.	N.D.	N.D.	0%
ZYFD030	*Microbacterium*	P11 BW	*Actinobacteria*	461.39 ± 314.22	N.D.	N.D.	N.D.	0.06 ± 0.08%
ZYFD042	*Microbacterium*	P11 BW	*Actinobacteria*	1236.21 ± 392.42	N.D.	N.D.		
ZYFB012	*Micrococcus*	P11 SW	*Actinobacteria*	1653.81 ± 277.64	N.D.	N.D.	N.D.	0.03 ± 0.04%
LZB055	*Phycicoccus*	ME3 SW	*Actinobacteria*	19.47 ± 10.57	N.D.	N.D.	N.D.	0%

a Rate of DMSP dependent DMS production in nmol DMS per mg protein per hour, average of three experiments with standard deviation in brackets.

b Y stands for positive activity to produce MeSH from DMSP. Due to the extremely volatile characterization of MeSH, the detected MeSH was unquantified.

c *N.D. stands for no detectable of DMS or MeSH production form DMSP, no ddd/dmdA gene detected under our experimental conditions or no Ddd/DmdA homologs detected from the genomes of taxonomically close strains to the ECS Ddd^+^ or Dmd^+^ strains*.

As expected, the majority of Ddd^+^ isolates were *Proteobacteria* of which *Alphaproteobacteria* dominated (66.67%), including many Roseobacters (accounting for 33.33% of all tested Ddd^+^ isolates), i.e., *Donghicola, Ruegeria, Oceanicola, Paracoccus*, and *Sulfitobacter* isolates; some non-Roseobacter strains, like strains of known Ddd^+^ genera *Ahrensia* and *Labrenzia*; and of many other unreported Ddd^+^ alphaproteobacterial genera *Aurantimonas, Erythrobacter, Jiella*, and *Oceanicaulis*. Surprisingly, only one gammaproteobacterial strain of *Halomonas* was shown to be Ddd^+^, while another *Halomonas* isolate was not.

Interestingly, the second largest group (12.28%) of cultivated Ddd^+^ isolates was *Actinobacteria*. These included *Agrococcus, Brevibacterium, Kytococcus, Microbacterium, Micrococcus*, and *Phycicoccus* genera which together accounted for 3.79% of total bacterial isolates. Until now, only members of *Rhodococcus* and *Clostridium* genera of gram-positive bacteria isolated from fresh water had been reported to be Ddd^+^ (Yoch et al., 2001). Furthermore, no functional *ddd* or *dmdA* genes have been identified in any gram-positive bacteria.

Under our conditions, strains of *Ahrensia, Oceanicola, Ruegeria, Sulfitobacter, Agrococcus, Micrococcus*, and *Microbacterium* showed similar or higher Ddd production rates (> 40 nmol DMS per mg protein per hour) when compared to the positive control strains, i.e., *Roseovarius nubinhibens* ISM, which contains functional *dddQ* and *dddP* genes (Todd et al., 2011), and *Oceanimonas doudoroffii* J495, which contains functional *dddP* and *dddD* genes (Curson et al., 2012). The other Ddd^+^ isolates showed relatively low Ddd production levels (Table 2).

In contrast to the high proportion isolates shown to be Ddd^+^, only two alphaproteobacterial strains, *Donghicola* sp. ZYFB040 (a Roseobacter) and *Erythrobacter* sp. LZB006, showed detectable Ddm production activity when tested with 1 μM DMSP concentration (Table 2). To exclude the possibility that DMSP added to the ECS isolates was insufficient to produce detectable MeSH levels, incubations with higher DMSP concentrations were conducted (0.5 and 5 mM). When exposed to higher DMSP levels, most strains likely to contain *dmdA* (i.e., *dmdA* was amplified from their genomic DNA or closely related strains have *dmdA* homologs in their genomes, see below) showed detectable MeSH production, including *Rhodobacteraceae* strains *Loktanella* sp. LZD013, *Oceanicola* LZD026, *Ruegeria* ZYFB035 and *Sulfitobacter* LZD018 and LZD014 (Table 2; Table S2). These strains were considered as DMSP demethylating bacteria. However, alphaproteobacterial strains of *Aurantimonas* ZYFD019 and *Henriciella* ZYFB017, and the gram-positive strains of *Mycobacterium* LZB054 and ZYFD013 (which were shown to contain *dmdA* by PCR) had no Ddm^+^ activity even with the increased DMSP concentrations. We tried to quantify the disappearance of DMSP of these strains in addition to the production of MeSH, but only *R. nubinhibens* ISM, the positive control, showed a statistically significant reduction in DMSP levels (*P* < 0.05) (Table S9). It is possible that these cells were degrading very low levels of DMSP via demethylation, but the MeSH produced from DMSP was quickly incorporated into biomass during the incubation, leaving a MeSH concentration below the MeSH detection limit used here. Thus, the absence of detectable DMSP dependent MeSH production does not necessarily indicate that a strain cannot demethylate DMSP. A similar situation could exist for Ddd in bacteria where DMS produced from DMSP is rapidly transformed, e.g., oxidized to dimethylsulfoxide. It would be interesting to see if the *dmdA* and/or *ddd* genes are functional and/or transcribed in strains that contain them but which show no detectable Ddd or Ddm phenotype.

To further investigate the potential molecular mechanisms of the Ddd and Ddm production in our isolates, we used published *dddD, dddL, dmdA* primers (Raina et al., 2009; Varaljay et al., 2010) and our newly designed and ratified *dddP* degenerate primers (Table S4) to screen for the presence of these functional DMSP lyase and demethylase genes in all representative strains (Table 2; Table S2). *dddL* homologs were amplified from genomic DNA of Ddd^+^ *Oceanicola* sp. LZD010 and LZD026, *Sulfitobacter* sp. LZD014 and *Labrenzia* sp. LZB033, displaying 52.90 to 100% amino acid identity to the cupin-containing DddL from *Sulfitobacter* EE36 (Curson et al., 2008). As expected, *dddP* homologs were amplified from Ddd^+^ *Sulfitobacter* sp. LZD018, *Ruegeria* sp. ZYFB035 and *Donghicola* sp. ZYFB040 with 79.68 to 88.24% amino acid identity to *R. nubinhibens* ISM DMSP lyase DddP (Todd et al., 2009). *Labrenzia* sp. LZB033 possessed DddP homologs, with 35.16% identity to *O. doudoroffii* J495 DddP2 (Curson et al., 2012). For the other isolates, no *ddd* gene could be amplified by the primers we used.

When probing the ECS isolates for DMSP demethylase gene *dmdA* using the published universal *dmdA* primer sets dmdAU (Varaljay et al., 2010), only *Loktanella* sp. LZD013, a strain that showed no detectable Ddm^+^ activity, gave the correct PCR product (Table S2). Given far more of the isolates were expected to contain *dmdA*, primers specifically targeting *dmdA* subclades (A/1, A/2, B/3, E/2) were also used (Varaljay et al., 2010). Using these primers, 12.28% of the representative isolates were shown to contain *dmdA* and thus the genetic potential to demethylate DMSP (Table S2). *dmdA* A/2 amplicons likely encoding functional enzymes were detected in *Oceanicola, Loktanella, Sulfitobacter*, and *Henriciella* isolates, which are genera of *Rhodobacteraceae*, and surprisingly, *dmdA* A/2 was also present in one actinobacterium of the genus *Mycobacterium*. Indeed, a recent metagenomic study suggested that some *Actinobacteria* can catabolize DMSP via the demethylation pathway and contain *dmdA* (Mizuno et al., 2015). Gammaproteobacterial E/2 subclade-targeting primers gave no PCR products with any g*ammaproteobacterial* isolate but did from one alphaproteobacterial *Aurantimonas* strain and another actinobacterium of the genus *Mycobacterium*.

Using BLASTp, we interrogated the available genomes of closely related strains that are in the same genera as our 57 representative strains with ratified Ddd lyase sequences (Table S3). DddD, DddL, DddP, DddQ, and DddW homologs were found in many of the alphaproteobacterial strains (Table S2). However, no known Ddd homologs were identified in the genomes of bacteria most closely related to the Ddd^+^ actinobacterial isolates or alphaproteobacterial *Erythrobacter, Aurantimonas, Jiella*, and *Oceanicaulis* isolates. DmdA homologs were present in sequenced genomes of *Ahrensia, Labrenzia, Oceanicola, Ruegeria*, and *Sulftobacte*r strains, but none of our isolates in these genera showed detectable levels of Ddm^+^ activity.

### Bacterial Community Structure

**Figure 3:**
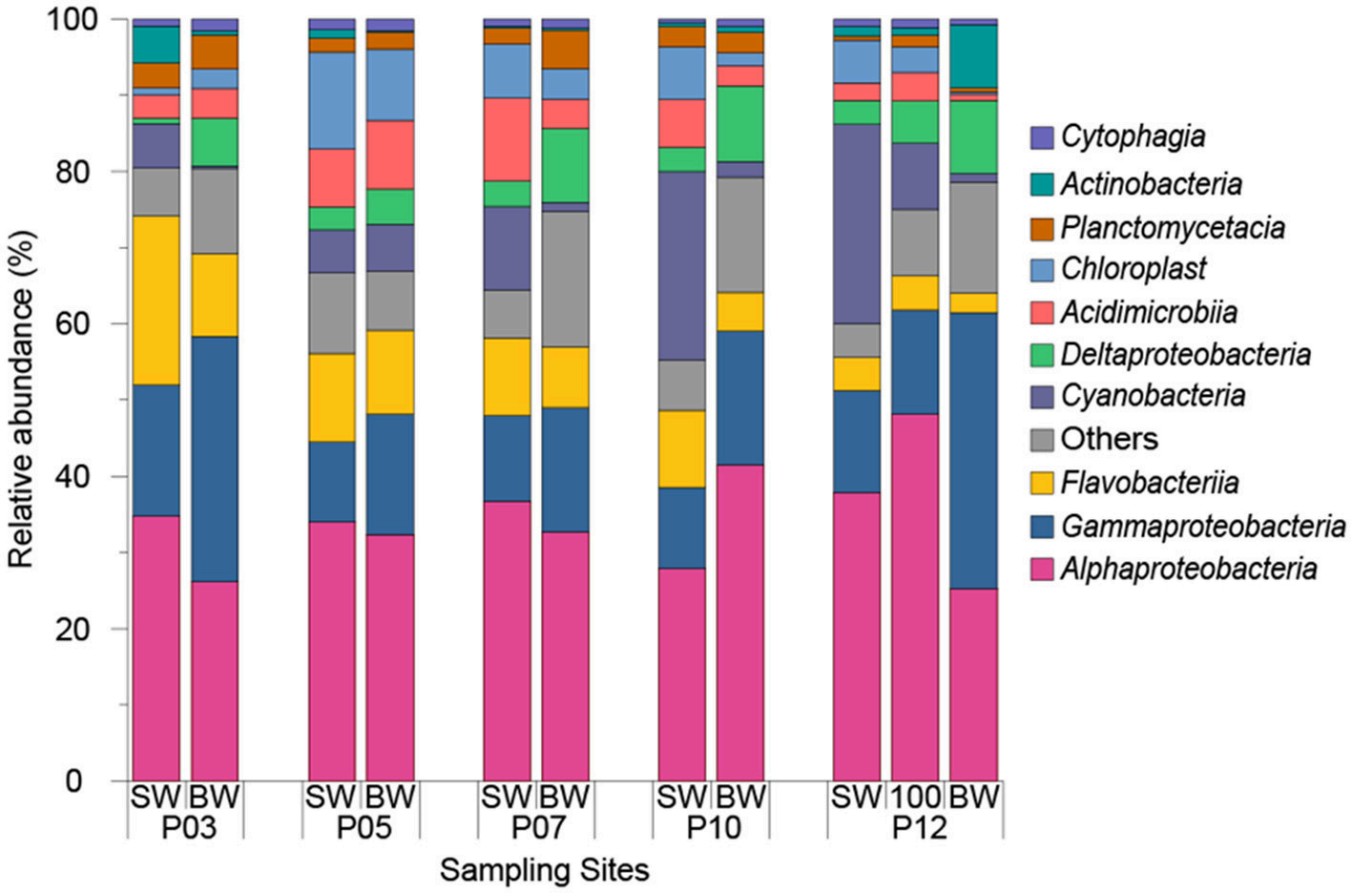
Relative abundance of top 10 dominant classes in seawater samples from 2015 cruise. “SW” stands for surface seawater, “BW” stands for bottom seawater.

Microbial community analysis was carried out by analyzing the diversity of 16S rRNA genes in SW and BW samples from five ECS sites across a transect from inshore to offshore waters. Based on 97% nucleotide identity level at the 16S rRNA gene level, a total of 3089 operational taxonomic units (OTUs) were obtained from 11 seawater samples. In all seawater samples, *Alphaproteobacteria* (34.31 ± 6.46%) and *Gammaproteobacteria* (17.70 ± 8.19%) were the most abundant classes (Figure 3).

The ECS *Alphaproteobacteria* largely comprised SAR11 clade and *Rhodobacterales* bacteria which together constituted 79.09 ± 5.25% of total *Alphaproteobacteria*. SAR11 dominated in almost all sampling sites (22.40 ± 6.88% of the total bacteria) except P03 where *Rhodobacterales* constituted 26.36% and 12.92% of total bacteria in SW and BW respectively. The relative abundance of *Rhodobacterales* showed positive correlations with DMS, DMSPt, and DMSPp concentrations (*r* = 0.89, *P* < 0.01), and Chl *a* concentration (*r* = 0.70, *P* < 0.05). Of all annotated *Rhodobacterales*, the genera *Ruegeria, Sulfitobacter, Paracoccus*, and *Labrenzia*, which we find to be Ddd^+^, represented on average 0.84 ± 0.57%, 0.70 ± 0.41%, 0.70 ± 0.59%, and 0.05 ± 0.04% of the total bacteria (Table 2). The genus *Roseovarius*, a member of which is also known to catabolize DMSP (González et al., 2003), was also identified and represented 0.63 ± 0.43% of the total bacteria. Consistent with the cultivation results, the above Ddd^+^ genera represented a large proportion (19.2% to 54.1%) of total *Rhodobacteraceae*. The Ddd^+^ genus *Aurantimonas* was found in most samples (represented 0.04 ± 0.01% of total bacteria) except P05 and BW of P03. *Erythrobacter*, which had Ddd^+^ representatives, made up 0.01 to 0.80% of total bacteria (Table 2). Although not isolated in this study, the most abundant genus of SAR11 was *Candidatus* Pelagibacter, representing 59.17 ± 17.89% of SAR11 clade (10.94 ± 5.65% of total bacteria). SAR11 bacteria contain *dmdA* and demethylate DMSP (Howard et al., 2006) and many SAR11 bacteria also contain *dddK* and cleave DMSP (Sun et al., 2016).

Of ECS *Gammaproteobacteria, Oceanospirillales* (7.37 ± 3.78% of total bacteria) and *Alteromonadales* (4.70 ± 3.67% of total bacteria) were the dominant orders representing 67.76 ± 15.73% of total *Gammaproteobacteria*. Correspondingly, *Halomonas* from *Oceanospirillales* and *Alteromonas* from *Alteromonadales* were the most abundant genera representing 0.08% to 1.33% (1.11% on average) and 0.42% to 3.44% (2.03% on average) of total bacteria, respectively. *Halomonas* representatives from both our ECS bacterial isolates and an isolate from the macroalgae *Ulva lactuca* are known to have Ddd^+^ activity (Todd et al., 2010). However, to our knowledge no *Alteromonas* isolates have been shown to catabolize DMSP or contain know *ddd* or *dmdA* genes.

*Cyanobacteria* which are known to import DMSP (Vila-Costa et al., 2006), were abundant in SW samples (5.56% to 26.16%) and were positively correlated with longitude (*r* = 0.94, *P* < 0.05). As expected, their abundance decreased in BW samples (ranged from 0.37% to 6.20%) with decreased light levels. At the genus level, *Synechococcus* made up 9.35 ± 7.58% of total bacteria in SW samples and was the second most abundant genera. To our knowledge, no *Cyanobacteria* has been shown to catabolize DMSP. Only one sequenced cyanobacterial strain, *Synechococcus* sp. KORDI-100, and one metagenome assembled *Synechococcus* genome (*Synechococcus* sp. TMED20) (Lei et al., 2017) have a putative DMSP lyase gene, this being *dddY*-like gene. Thus, *Cyanobacteria* are not believed to be significant DMSP catabolisers. In contrast to *Cyanobacteria, Deltaproteobacteria* were always more abundant in BW (8.01 ± 2.19%) compared to SW samples (2.67 ± 0.95%). Although no Ddd^+^ or Ddm^+^ *Deltaproteobacteria* were isolated in this study likely due to the isolation conditions, *Desulfovibrio acrylicus* is known to contain a DMSP lyase, likely DddY, and cleave DMSP (Der Maarel et al., 1996; Curson et al., 2011a).

### The Abundance and Diversity of *dmdA* and *dddP* in the ECS Samples

The *dmdA* and *dddP* genes are the most abundant environmental indicators of DMSP demethylation and cleavage (Curson et al., 2018) and qPCR primers targeting these genes have been designed (Varaljay et al., 2010). These primer sets were used to investigate the abundance of *dddP* and *dmdA* in ECS samples. It should be noted that the *dmdA* primer sets only target about half of known *dmdA* sequences.

**Figure 4:**
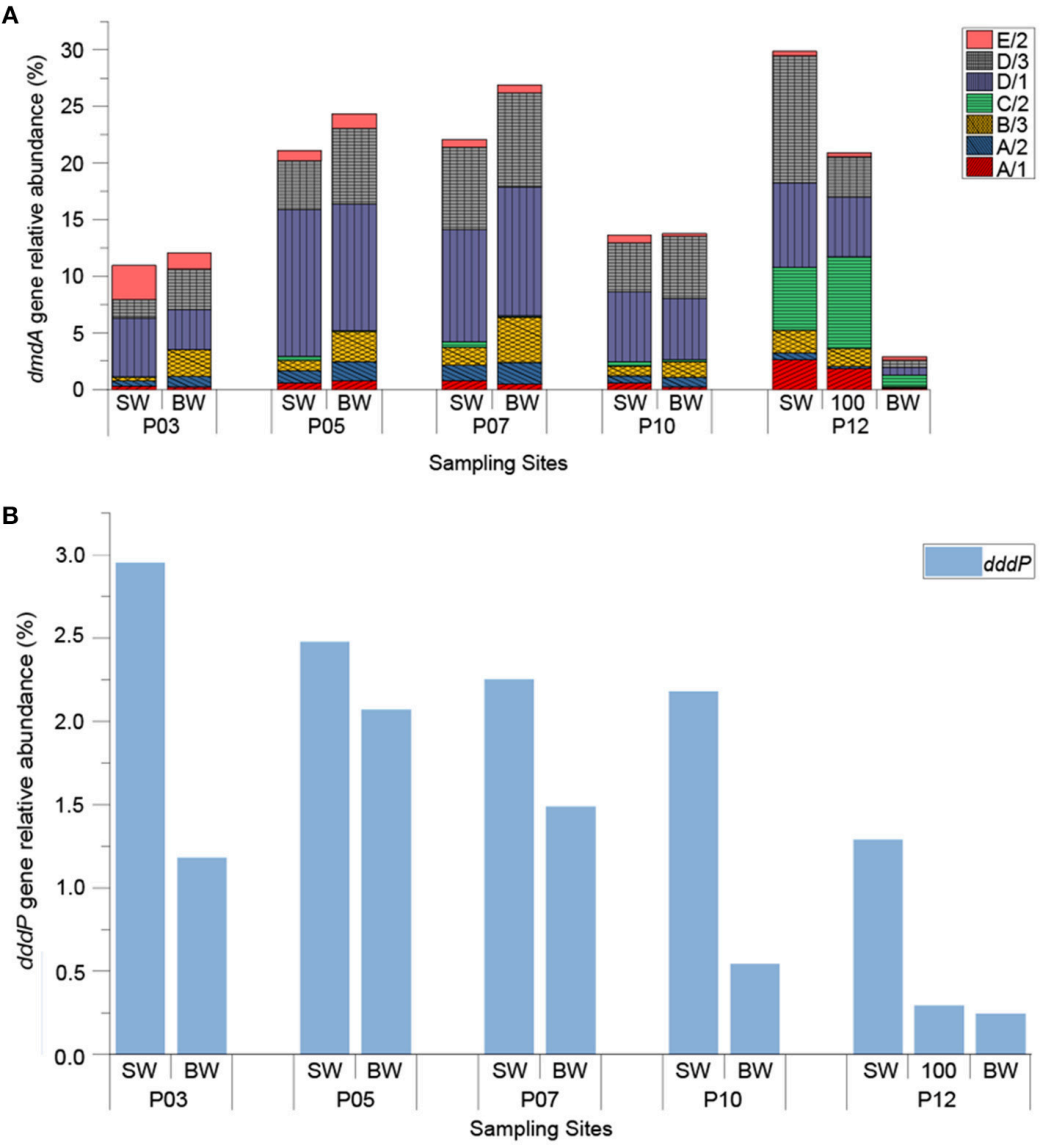
The relative abundance of bacterial DMSP-degrading genes in ECS seawater samples. The *dddP* and *dmdA* genes were normalized against bacterial 16S rRNA copy numbers. **(A)** Seven different subclades of *dmdA*; **(B)** *dddP*. “SW” stands for surface seawater; “BW” stands for bottom seawater; “100” stands for seawater of 100 m depth.

The relative abundance of DMSP-degrading genes (normalizing *ddd* and *dmdA* gene copy numbers to that of the bacterial 16S rRNA gene copy numbers) are shown in Figure 4. As expected, *dmdA* was abundant in the ECS seawaters, with the total relative abundances of all the *dmdA* subclades ranging from 2.89 to 29.88% (Figure 4A). Apart from BW sample of P12 (999 m in depth), the total relative abundance of *dmdA* subclades was 19.57% on average, and had no significant difference (*P* > 0.05) between the SW and BW samples, but was positively correlated with temperature (*r* = 0.72, *P* < 0.05) (Figure S2). Within samples excluding BW sample of P12, *dmdA* SAR11 subclades D/1 (2.51 × 10^6^ to 6.56 × 10^8^ copies L^−1^) and D/3 (1.69 × 10^6^ to 4.68 × 10^8^ copies L^−1^) were the most abundant; SAR11 subclade C/2 (6.89 × 10^5^ to 4.30 × 10^7^ copies L^−1^) was comparatively abundant in the offshore site P12, particularly in the SW and 100 m depth samples (with relative abundance of 5.56% and 8.12%, respectively). On average, the relative abundances of Roseobacter subclades A/1 and A/2 were 0.98% and 0.82%, respectively in SW samples, and 0.41% and 1.35%, respectively in BW samples. The relative abundance of *Gammaproteobacteria*-derived subclade E/2 showed a decreasing trend along the transection from inshore (3.04%) to offshore (0.36%), and negatively correlated with longitude (*r* = −0.75, *P* < 0.01) (Figure S2). The *dmdA* subclade B/3 that is represented by the SAR116 group member “*Candidatus* Puniceispirillum marinum” (Oh et al., 2010) was more abundant in BW samples (relative abundance of 2.61% on average) than in SW samples (relative abundance of 0.94% on average), with *P* < 0.05.

It was clear that the genetic potential to cleave DMSP via the DddP DMSP lyase is far less prominent in the ECS samples than that for DMSP demethylation (Figure 4B). This is expected and fits with previous metagenomic predictions (Moran et al., 2012). The abundance of *dddP* ranged from 1.91 × 10^4^ to 2.34 × 10^8^ copies L^−1^, with an average of 6.03 × 10^7^ copies L^−1^. Unlike *dmdA*, the *dddP* gene was significantly more abundant in all SW samples than in BW samples (*P* < 0.05). This fits with DMSP cleaving bacteria being most abundant in SW where DMSP concentrations are higher. The relative abundance of *dddP* was highest in the SW of P03 site (2.95%) and steadily decreased as the sites moved away from land (to 0.244% in BW of P12), showing a negative correlation with longitude (*r* = −0.7, *P* < 0.05), and a positive correlation with Chl *a* concentration (*r* = 0.68, *P* < 0.05) (Figure S2). This data implies that *dddP* may be more prominent in bacteria that closely associate with phytoplankton, like *Rhodobacteraceae*. DMS concentration in ECS samples positively correlated with the proportion of the *dddP* gene in the total DMSP-degrading genes (*dddP*/total *dmdA* + *dddP*) (*r* = 0.94, *P* < 0.01) (Figure S2). Although this value does not include other prominent DMSP lyase genes, correlation still implies the competing relationship between DMSP demethylation and cleavage.

**Figure 5:**
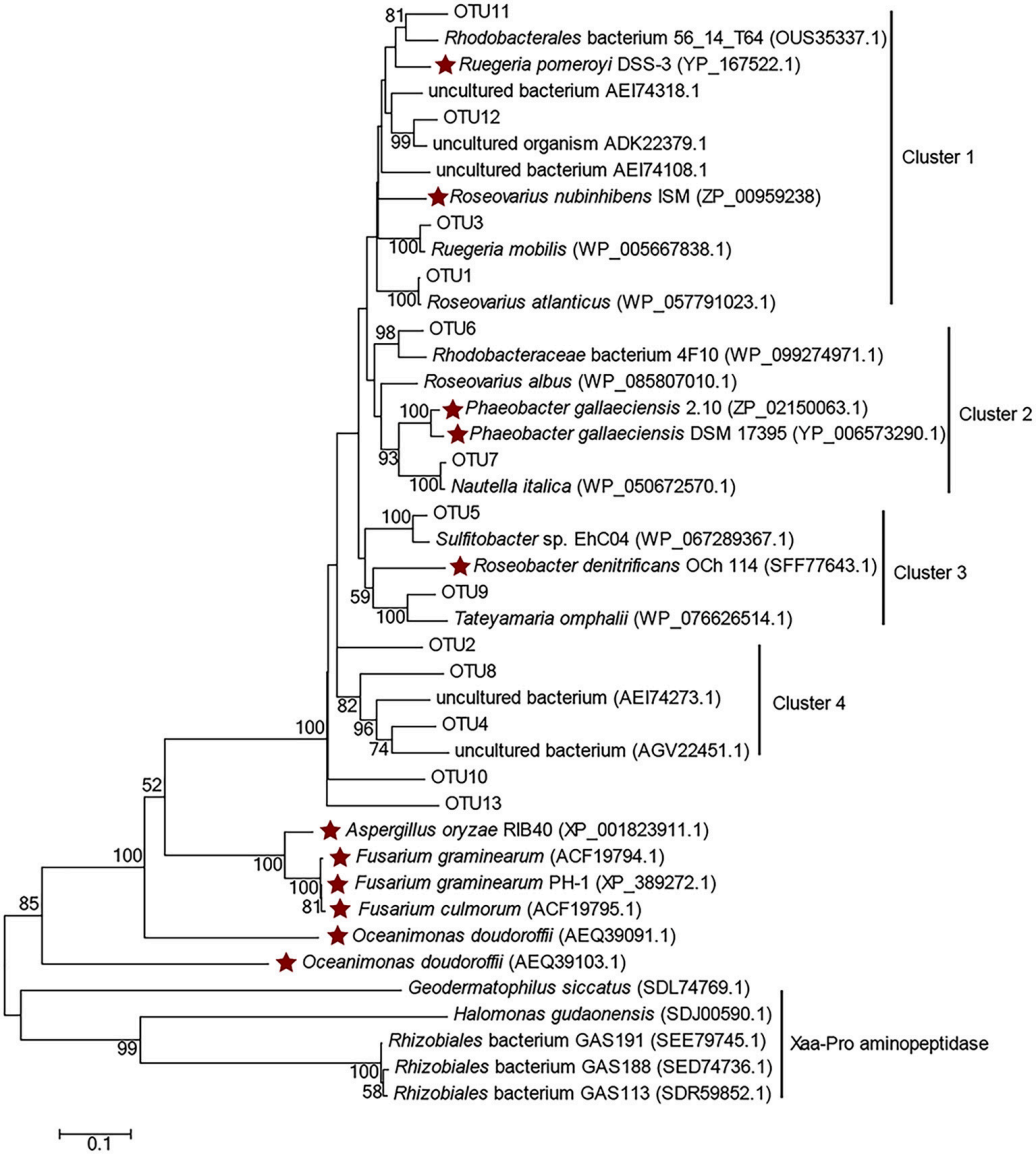
Amino acid tree of representative DddP OTU sequences and their top-hit sequences in GenBank. Xaa-Pro aminopeptidase sequences were used as the outgroup. Stars represent the DddPs which have been experimentally ratified to be functional. The neighbor-joining tree was made with the Passion model.

Clone libraries of *dddP* were constructed from samples of P11, ME3, P03, P05, P10, and P12 (except BW samples of ME3 and P12, since no PCR amplicon could be achieved). In total 314 clones were sequenced and classified into 13 OTUs (Table S6), among which OTU1, OTU2, and OTU3 were dominant. Most representative OTU sequences were clustered with functional DddP sequences of Roseobacter clade bacteria (Figure 5), except those affiliated in cluster 4 in which representative OTUs were clustered with DddP homologs from uncultured bacteria, including OTU2 (represented 31.53% of total sequences). This implies that uncultured bacteria, likely of the *Rhodobacteraceae*, make a significant contribution to bacterial DMSP cleavage in the ECS, highlighting the need to combine culture-dependent and -independent techniques.

## Discussion

Bacterial demethylation and lysis of DMSP are important and well-established components of the sulfur cycle in marine environment. This study found that a relatively high proportion (42.11%) of the tested cultivated heterotrophic bacteria had the capacity to cleave DMSP generating DMS. Of course, this is not necessarily reflecting the total bacterial community since the composition of the tested cultivable bacteria is not wholly reflecting the composition of the total cultivable isolates, and moreover, we cannot reliably predict the proportion of uncultivated Ddd^+^ bacteria via the methods used here. Curson et al. (2018) predicted that ~20% of total bacteria in the OM-RGC metagenomic dataset, mainly apportioned surface ocean bacteria, contain known *ddd* homologs and thus the genetic potential to cleave DMSP. Although this value is relatively lower than the culture-dependent work presented here, both methods confirmed that Ddd^+^ bacteria are abundant in the natural marine environment.

Many of the ECS Ddd^+^ isolates are of genera whose members have been demonstrated to cleave DMSP and contain known DMSP lyases, including the alphaproteobacterial *Ruegeria, Labrenzia, Donghicola, Sulfitobacter, Oceanicola*, and gammaproteobacterial *Halomonas* (Curson et al., 2008, 2011b). Every Ddd^+^ roseobacterial isolate and the *Labrenzia* isolate were found to contain either DddL and/or DddP DMSP lyases that were all very closely related to functionally ratified DMSP lyases (Curson et al., 2008; Todd et al., 2009). The *Ahrensia* DddL proteins predicted from the genome sequence of LZD062 (Liu et al., 2016) was only 46.98% to that of *Sulfitobacter* EE-36 (Curson et al., 2008), but was cloned and shown to encode a functional DMSP lyase by heterogenous expression in *Escherichia coli* BL21 (DE3) (140.01 ± 14.63 nmol DMS mg protein^−1^ h^−1^). These data extend our knowledge as to what constitutes a functional DddL DMSP lyase protein.

The Ddd^+^ alphaproteobacterial isolates in the genera of *Jiella* and *Aurantimonas* are in the same *Aurantimonadaceae* family as Ddd^+^ *Fulvimarina pelagi* that contains DddL (Curson et al., 2008). However, both *Jiella* and *Aurantimonas* isolates gave no products when probed for *dddL, dddP*, and *dddD* primers, thus we do not know the identity of the DMSP lyase in these isolates. The Ddd^+^ *Halomonas* isolate which could use DMSP as sole carbon source did not give a product with *dddD* or any other *ddd* primer sets used. Since *Halomonas* HTNK1 has *dddD*, uses DMSP as sole carbon source (Todd et al., 2010), and other sequenced bacteria of this genera contain DddP homologs, it is most likely that the *dddD* and/or *dddP* genes in the ECS *Halomonas* were not captured by the primer sets used. However, it cannot be ruled out that the ECS *Halomonas* isolate contains other known *ddd* genes, like *dddL, dddQ, dddY* or *dddW*, or even novel *ddd* gene(s).

Many Ddd^+^ isolates from ECS were of genera not reported or even suspected to catabolize DMSP, including *Erythrobacter* isolates in order *Sphingomonadales* and many gram-positive *Actinobacteria* isolates of the *Agrococcus, Brevibacterium, Kytococcus, Microbacterium, Micrococcus*, and *Phycicoccus* genera. This is the first report of any gram-positive marine actinobacteria making DMS from DMSP. None of the Ddd^+^ actinobacterial isolates gave any products with the *dddL, dddP* or *dddD* primer sets, nor were there any Ddd homologs in the sequenced genomes of closely related bacteria. We sequenced the genomes of two Ddd^+^ *Actinobacteria* (*Microbacterium* ZYFD042 and *Agrococcus* sp. LZB059, which grew well in MAMS media and also showed relatively high Ddd^+^ levels), finding only *dddD*-like gene in the genome of ZYFD042, with 35.64% amino acid similarity to DddD of *Marinomonas* sp. MWYL1 (Todd et al., 2007). When cloned and expressed it in *E. coli*, this DddD-like enzyme did not cleave DMSP. Thus, it is most likely these bacteria contain novel DMSP lyase enzymes. Future molecular work is required to identify the novel DMSP lyase gene(s).

In comparison to e.g., SAR11 and Roseobacter bacteria, Ddd^+^ genera of *Actinobacteria* were not abundant in the ECS samples and were not detected in all samples (Table 2). *Brevibacterium* was only seen in SW of P05 (0.01%) and BW of P12 (0.02%). *Microbacterium* was found in most samples (representing 0.06 ± 0.08% of total bacteria) except in the BW of P03, P05, and P07. *Micrococcus* was only found in BW and comprised less than 0.01% of total bacteria on average. It is unlikely these Ddd^+^ *Actinobacteria* constitute major contributors to DMS production in these environments. Further molecular work on these bacteria is required to test these hypotheses.

Quantification of *dmdA* subclade genes (normalized to 16S rRNA gene copy numbers) indicated their abundant distribution in both SW (19.53 ± 6.70%) and BW (16.00 ± 8.73%) ECS samples (Figure 4A), although at lower levels than previously reported (~60% of bacteria) in the GOS metagenomic data (Howard et al., 2008). This discrepancy may partly be due to the limitation of primer sets that do not cover all the *dmdA* subclades and/or to the selection of 16S rRNA gene as the normalizing gene, since some bacterial genomes have multiple copies of 16S rRNA gene (Cui et al., 2015). Nevertheless, this work confirms that the genetic potential for the bacterial DMSP demethylation is prevalent in the ECS. Culture-dependent work showed that in the majority of cases where *dmdA* was found in a bacterium, the said bacterium had detectable MeSH production when grown in the presence of DMSP. However, this was not always the case. Further work involving RNA and/or proteomics on the environmental and pure cultures is required to establish why bacteria, e.g., *Aurantimonas* ZYFD019, *Henriciella* ZYFB017, and the gram-positive strains of *Mycobacterium* LZB054 and ZYFD013, that contain *dmdA* homologs but do not generate MeSH from DMSP.

In the transect of ECS studied here, we found that the concentrations of Chl *a*, DMS, and DMSP (in the surface seawater) gradually decreased with the distance to the land (longitude), while the salinity and temperature showed increasing trends (Table 1; Figure S2), which provided us natural environmental gradients to study correlations between bacterial DMSP degradation and environmental factors. The predicted major DMSP-degrading bacterial groups, *Rhodobacteraceae* and SAR11, showed contradictory changes in relative abundances based on the 16S rRNA gene amplicon sequencing data (Figure S2H) and had different correlations with Chl *a*, DMSPt, and DMS concentrations (Figure S2). *Rhodobacteraceae* are normally abundant in coastal environments and associated with oceanic algal blooms (Gonzalez and Moran, 1997; Simo et al., 2000; Moran et al., 2004). Their positive correlations with DMS and DMSPt concentrations implied that *Rhodobacteraceae* might be the major DMS producer from DMSP in the ECS study sites. However, since some phytoplankton directly cleave DMSP to DMS, the correlations might also be due to the association of *Rhodobacteraceae* and phytoplankton (Figures S2I–K). SAR11 bacteria are widely distributed in marine seawaters and particularly abundant in the open ocean where relatively low DMSP concentrations exist (Giovannoni, 2005, 2017). SAR11 bacteria lack a sulfur reductase and require reduced organosulfur compounds, like DMSP, in surrounding seawater (Tripp et al., 2008). The negative correlation between SAR11 and DMS, DMSPt concentrations implies that SAR11 are not significant contributions to DMS production from DMSP in the studied areas of the ECS (Figures S2L–N). Although, it should be noted that standing stock concentrations of DMSP and DMS are not always good indicators of activity. The positive correlation between DMS and DMSP concentrations with the total relative abundance of *dmdA* SAR11 subclade D1 and D3 (*r* = 0.72, *P* < 0.05) (Figure S2O) implies that SAR11 might degrade DMSP mainly through the demethylation pathway.

Overall, using culture-dependent methods, we identify DMSP catabolizing bacterial taxa in the ECS, many of which are novel gram-positive DMSP-cleaving *Actinobacteria*. This further extends the phylogenetic territory of marine microorganisms that can carry out this globally influential process. The work provides necessary model organisms to discover novel pathway(s) and gene(s) for DMSP degradation. Also, through culture-independent methods, we find a high proportion of ECS bacteria have the genetic potential to catabolize DMSP, and highlight heterotrophic *Alphaproteobacteria*, particularly SAR11 and Roseobacter bacteria, as key degraders. Further work studying the activity and regulation of key DMSP lyase and demethylase genes in this environment will shed light on the relative contribution of these two competing pathways.

## Author Contributions

X-HZ and JT designed the experiments and wrote the manuscript. JingL and JL collected samples, performed experiments and wrote the manuscript. S-HZ, G-PY performed experiments (DMS measurement on GC, Chl *a* concentration measurement). JincL helped to perform experiments (qPCR and clone library construction). HL conducted BLASTp analysis of DmdA and Ddd homologs in genomes of taxonomically related strains to Ddd^+^ bacterial isolates. DS performed the PCR amplicon of *ddd* and *dmdA* from genomic DNA of all the tested bacterial isolates.

### Conflict of Interest Statement

The authors declare that the research was conducted in the absence of any commercial or financial relationships that could be construed as a potential conflict of interest.
