# Supplementary material for: Novel Insights Into Bacterial Dimethylsulfoniopropionate Catabolism in the East China Sea
Source: Front Microbiol. 2018 Dec 21;9:3206. doi: 10.3389/fmicb.2018.03206 (PMC6309047; doi:10.3389/fmicb.2018.03206)
Supplement: Supplementary file 1 [file Data_Sheet_1.doc]

# *Supplementary Material*

# Novel insights into bacterial dimethylsulfoniopropionate catabolism in the East China Sea

**Jingli Liu^1,2^†, Ji Liu^1,2^†, Sheng-Hui Zhang^3^,** **Jinchang Liang^1^, Heyu Lin^1^, Delei Song^1^, Gui-Peng Yang^3,4^, Jonathan D. Todd^2*^, Xiao-Hua Zhang^1,4*^**

^1^ College of Marine Life Sciences, Ocean University of China, Qingdao, China.

^2^ School of Biological Sciences, University of East Anglia, Norwich Research Park, Norwich, UK.

^3^ College of Chemistry and Chemical Engineering, Ocean University of China, Qingdao, China.

^4^ Laboratory for Marine Ecology and Environmental Science, Qingdao National Laboratory for Marine Science and Technology, Qingdao, China.

†These authors contributed equally to this work.

***Correspondence:**

Dr. Xiao-Hua Zhang, [xhzhang@ouc.edu.cn](mailto:xhzhang@ouc.edu.cn); Dr. Jonathan D. Todd, jonathan.todd@uea.ac.uk

## Supplementary Figures


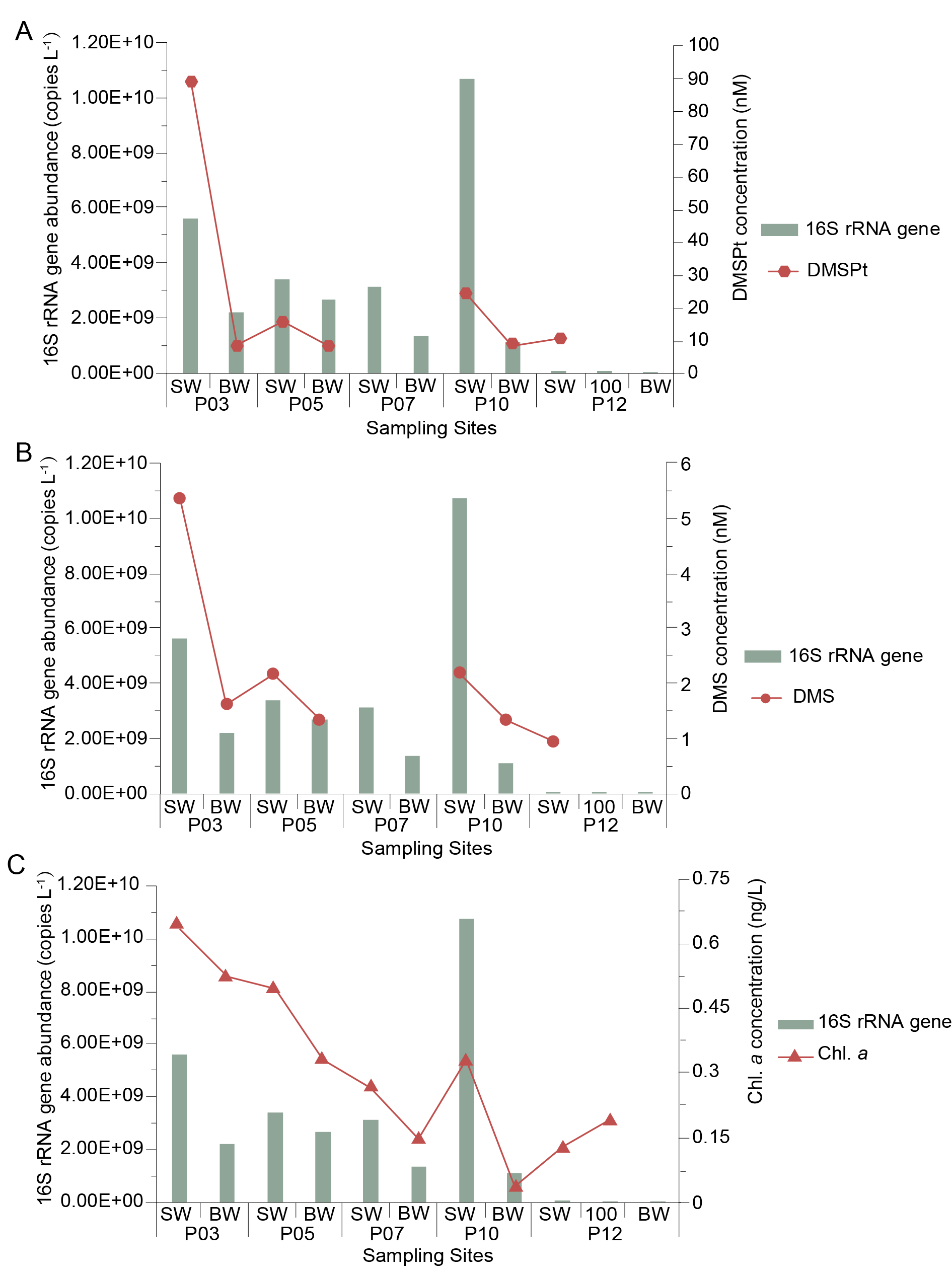


**Figure S1** DMS and Chl. *a* concentrations and abundance of 16S rRNA gene quantified by qPCR. Bar: gene copies number of 16S rRNA gene, solid pentagon: DMSPt concentration; circles: DMS concentration; solid triangles: Chl. *a* concentration.


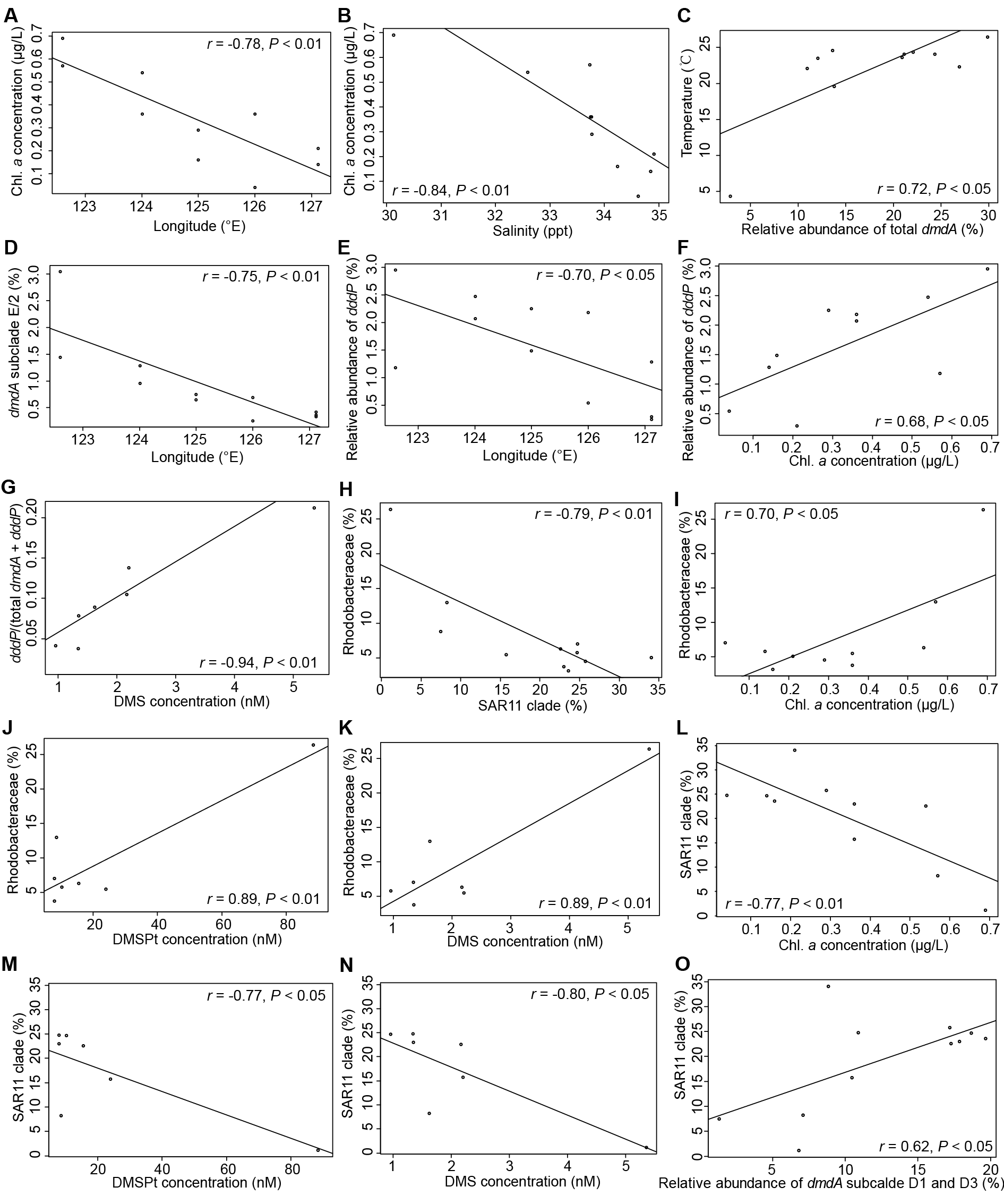


**Figure S2** Correlation analysis of the environmental parameters, relative abundance of DMSP-degrading genes and 16S rRNA genes from amplicon sequencing data. **(A)** Chl. *a* concentration and longitude. **(B)** Chl. *a* concentration and salinity. **(C)** the relative abundance of total *dmdA* genes and temperature. **(D - E)** the variation of the relative abundance of *dmdA* subclade E/2 and *dddP* gene along distance to land/longitude. **(F)** the relative abundance of *dddP* and Chl. *a* concentration. **(G)** the value of *dddP*/(total *dmdA* + *dddP*) and DMS. **(H – N)** Correlations between relative abundance of SAR11 clade and *Rhodobacteraceae* in 16S rRNA gene amplicon sequencing data, and their variation trend with Chl. *a*, DMSPt and DMS. **(O)** positive correlation between relative abundance of SAR11 and total relative abundance of *dmdA* subclade D1 and D3.


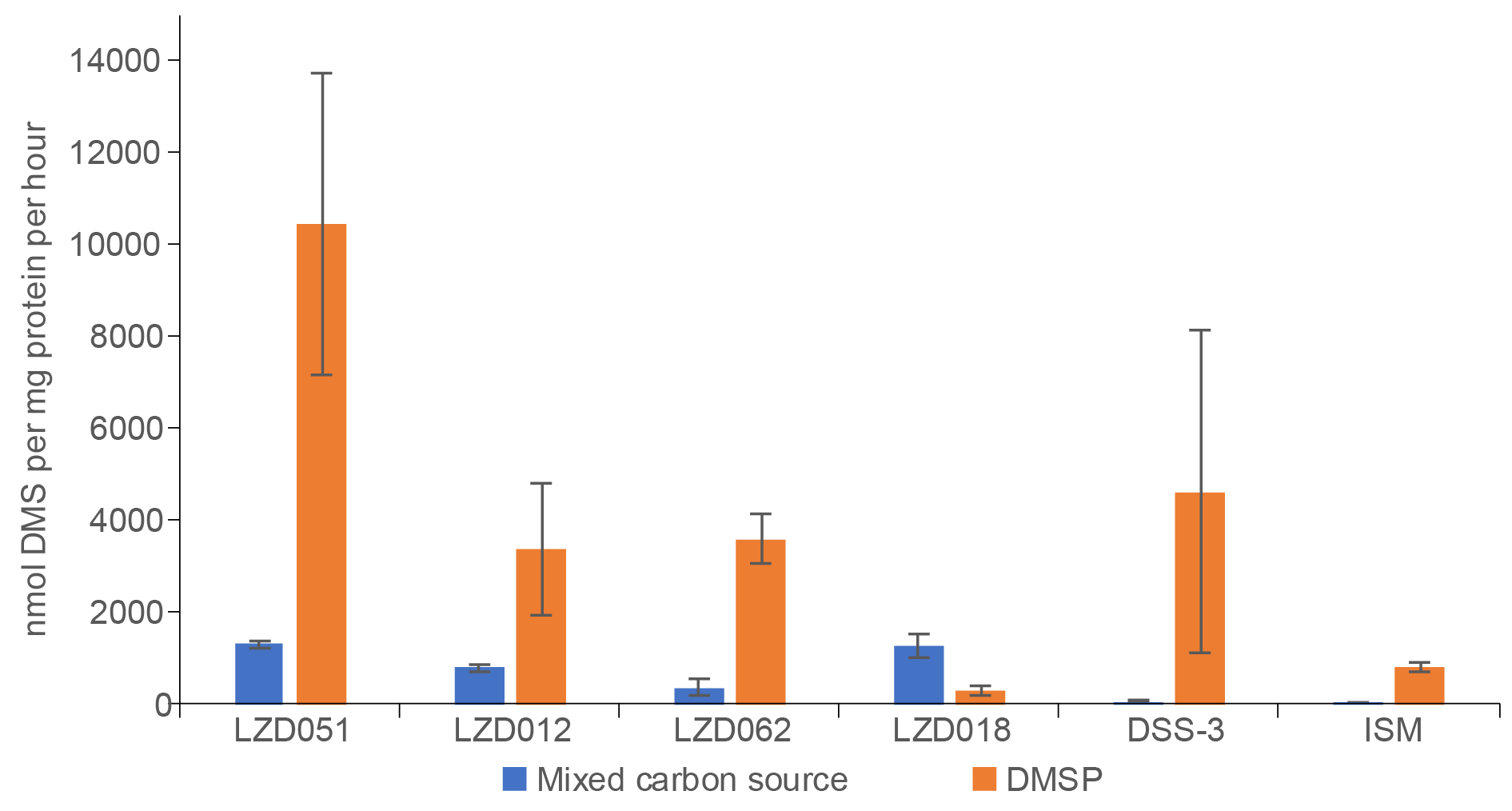


**Figure S3** DMS production from DMSP of strains that can use DMSP as sole carbon source under conditions with or without extra mixed carbon source (2mM glucose, 2mM succinate, 2mM sucrose, 2mM pyruvic acid sodium salt and 2mM glycerol).

## Supplementary Tables

**Table S1** Composition of modified MAMS medium*

| Solution& | Components (g/L) |
| --- | --- |
| 1 | NaCl (25.0), (NH_4_)_2_SO_4_ (1.0), CaCl_2_·H_2_O (0.2) |
| 2 | MgSO_4_·7H_2_O (1.0), FeSO_4_·7H_2_O (0.002), Na_2_MoO_4_ (0.002) |
| 3 | KH_2_PO_4_ (3.6), K_2_HPO_4_·3H_2_O (6.0) |
| 4 | Trace metal solution# |
| 5 | Vitamin solution† |

*The MAMS medium was modified from that of (Raina et al., 2009).

&Solution 1 was autoclaved at 121°C for 20 min; Solutions 2-5 were filter-sterilised separately. pH was adjusted to 7.6.

#1 ml/L (Balch et al., 1979).

†10 ml/L (Steinsbu et al., 2010).

**Table** **S2**. DMSP dependent DMS (Ddd^+^) and/or MeSH (Ddm^+^) production ability, PCR check of DMSP degrading genes of the 57 representative strains selected from difference genera and DMSP degradation enzyme homologues in the genomes of closely related reference strains.

| Representative strain | Closest taxonomically related genus | Strain source | DMSP-dependent DMS/MeSH production | *ddd* and *dmdA* by normal or degenerate PCR | Reference genome of closely related strain(s) from the same genus | GenBank accession number of genome | DMSP degradation enzyme homologue | Accession number of the homologue | E-value | Identity |
| --- | --- | --- | --- | --- | --- | --- | --- | --- | --- | --- |
| ZYFB010 | *Aeromicrobium* | P11 SW | N.D. | N.D. | *Aeromicrobium marinum* DSM 15272  *Aeromicrobium massiliense* JC14 | GCA_000160775.2  GCA_000312105.1 | N.D.  N.D. | -  - | -  - | -  - |
| LZB059 | *Agrococcus* | ME3 SW | DMS | N.D. | *Agrococcus lahaulensis* DSM 17612  *Agrococcus pavilionensis* RW1 | GCA_000160775.2  GCA_000400485.1 | N.D.  N.D. | -  - | -  - | -  - |
|  |  |  |  |  |
| LZD051  LZD062 | *Ahrensia*  *Ahrensia* | ME3 BW  ME3 BW | DMS  DMS | N.D.  N.D. | *Ahrensia kielensis* DSM 5890  *Ahrensia* sp. 13_GOM-1096m  *Ahrensia* sp. R2A130 | GCA_000374465.1  GCA_000620605.1  GCA_000179775.1 | DddD  N.D.  DmdA  DddP | WP_018690356  -  EFL87629  EFL90704 | 0  -  4e-91 to 2e-81  7e-176 to 2e-119 | 51.49%–67.31%  -  40.06%–42.99%  45.80%–58.92% |
| ZYFD019 | *Aurantimonas* | P11 BW | DMS | *dmdA E/2* | *Aurantimonas manganoxydans* SI85-9A1  *Aurantimonas coralicida* DSM 14790 | GCA_000153465.1  GCA_000421645.1 | N.D.  N.D. | -  - | -  - | -  - |
| LZB012  LZD038 | *Bacillus*  *Bacillus* | ME3 SW  ME3 BW | N.D.  N.D. | N.D.  N.D. | *Bacillus aquimaris*  *Bacillus pumilus* SAFR-032 | GCA_000935355.1  GCA_000017885.1 | N.D.  N.D. | -  - | -  - | -  - |
| LZD025  LZD055 | *Brevibacterium*  *Brevibacterium* | ME3 BW  ME3 BW | DMS  N.D. | N.D.  N.D. | *Brevibacterium senegalense*  *Brevibacterium album* DSM 18261 | GCA_000285835.1  GCA_000426445.1 | N.D.  N.D. | -  - | -  - | -  - |
| LZB056 | *Cellulomonas* | ME3 SW | N.D. | N.D. | *Cellulomonas flavigena* DSM 20109  *Cellulomonas fimi* ATCC 484 | GCA_000092865.1  GCA_000212695.1 | N.D.  N.D. | -  - | -  - | -  - |
| LZB003  LZD024 | *Citromicrobium*  *Citromicrobium* | ME3 SW  ME3 BW | N.D.  N.D. | N.D.  N.D. | *Citromicrobium bathyomarinum* JL354  *Citromicrobium* sp. JLT1363 | GCA_000176355.1  GCA_000186705.2 | N.D.  N.D. | -  - | -  - | -  - |
| ZYFB006 | *Croceibacter* | P11 SW | N.D. | N.D. | *Croceibacter atlanticus* HTCC2559 | GCA_000196315.1 | N.D. | - | - | - |
| ZYFB040 | *Donghicola* | P11 SW | DMS and MeSH | *dddP* | *Donghicola xiamenensis* DSM 18339  *Donghicola* sp. S598 | GCA_000429365.1  GCA_000308135.1 | N.D.  DddW | -  WP_017468368 | -  1e-42 | -  59.17% |
| LZB006  LZD016 | *Erythrobacter*  *Erythrobacter* | ME3 SW  ME3 BW | DMS and MeSH  DMS | N.D.  N.D. | *Erythrobacter litoralis* HTCC2594  *Erythrobacter longus* | GCA_000013005.1  GCA_000715015.1 | N.D.  N.D. | -  - | -  - | -  - |
| LZD012  LZD049 | *Halomonas*  *Halomonas* | ME3 BW  ME3 BW | DMS  N.D. | N.D.  N.D. | *Halomonas elongata* DSM 2581  *Halomonas campaniensis*  *Halomonas zhanjiangensis* DSM 21076 | GCA_000196875.1  GCA_000696485.1  GCA_000377665.1 | DddP1  DddP2  N.D.  N.D. | CBV41007  CBV41340  -  - | 8e-108 to 2e-101  5e-116 to 5e-105  -  - | 41.00%–42.56%  40.87%–45.03%  -  - |
| ZYFB003  ZYFB017 | *Henriciella*  *Henriciella* | P11 SW  P11 SW | N.D.  N.D. | N.D.  *dmdA A/2* | *Henriciella marina* DSM 19595 | GCA_000376805.1 | N.D. | - | - | - |
| ZYFB039 | *Hyphomonas* | P11 SW | N.D. | N.D. | *Hyphomonas oceanitis* SCH89  *Hyphomonas neptunium* ATCC 15444 | GCA_000685295.1  GCA_000013025.1 | N.D.  N.D. | -  - | -  - | -  - |
| LZB061 | *Janibacter* | ME3 SW | N.D. | N.D. | *Janibacter hoylei* PVAS-1  *Janibacter sp.* HTCC2649 | GCA_000297495.1  GCA_000152705.1 | N.D.  N.D. | -  - | -  - | -  - |
| LZB041 | *Jiella* | ME3 SW | DMS | N.D. | - | - | - | - | - | - |
| LZB010 | *Kytococcus* | ME3 SW | DMS | N.D. | *Kytococcus sedentarius* DSM 20547 | GCA_000023925.1 | N.D. | - | - | - |
| LZB033 | *Labrenzia* | ME3 SW | DMS | *dddL, dddP* | *Labrenzia alexandrii* DFL-11  *Labrenzia aggregata* IAM 12614 | GCA_000158095.1  GCA_000168975.1 | DmdA1  DmdA2  DddL  DddP1  DddP2  DddL  DddP | EEE47829  EEE44080  EEE47811  EEE43511  EEE44699  EAV43167  EAV46154 | 2e-180 to 5e-113  6e-99  1e-142 to 5e-71  6e-167 to 1e-124  8e-122  7e-165 to 1e-66  9e-119 to 2e-102 | 43.21%–66.39%  43.82%  49.77%–80.26%  48.09%–56.31%  45.73%  50.95 %–95.63%  41.33%–45.11% |
| ZYFB002  LZD017 | *Leeuwenhoekiella*  *Leeuwenhoekiella* | P11 SW  ME3 BW | N.D.  N.D. | N.D.  N.D. | *Leeuwenhoekiella blandensis* MED217 | GCA_000152985.1 | N.D. | - | - | - |
| LZD013 | *Loktanella* | ME4 BW | MeSH | *dmdA A/2* | *Loktanella cinnabarina* LL-001  *Loktanella hongkongensis* DSM 17492  *Loktanella vestfoldensis* SKA53 | GCA_000466965.1  GCA_000600975.1  GCA_000152785.1 | DddD  DddW  DmdA  DddL | WP_021695383  EYD72657  EAQ06035  WP_007204311 | 0  2e-44  3e-106  5e-119 to 4e-62 | 54.23%–72.13%  59.17%  43.73%  49.52%–73.95% |
| LZD057  ZYFB019 | *Maribacter*  *Maribacter* | ME5 BW  P11 SW | N.D.  N.D. | N.D.  N.D. | *Maribacter antarcticus* DSM 21422  *Maribacter forsetii* DSM 18668 | GCA_000621125.1  GCA_000744105.1 | N.D.  N.D. | -  - | -  - | -  - |
| ZYFB005 | *Maricaulis* | P11 SW | N.D. | N.D. | *Maricaulis maris* MCS10  *Maricaulis* sp. JL2009 | GCA_000014745.1  GCA_000412185.1 | N.D.  N.D. | - | - | - |
| LZD059  LZD060 | *Marinobacter*  *Marinobacter* | ME3 BW  ME4 BW | N.D.  N.D. | N.D.  N.D. | *Marinobacter algicola* DG893  *Marinobacter hydrocarbonoclasticus* VT8  *Marinobacter adhaerens* HP15 | GCA_000170835.1  GCA_000015365.1  GCA_000166295.1 | N.D.  N.D.  N.D. | -  -  - | -  -  - | -  -  - |
| ZYFD030  ZYFD042 | *Microbacterium*  *Microbacterium* | P11 BW  P11 BW | DMS  DMS | N.D.  N.D. | *Microbacterium hominis*  *Microbacterium trichothecenolyticum*  *Microbacterium hydrocarbonoxydans*  *Microbacterium azadirachtae* | GCA_000813805.1  GCA_000956465.1  GCA_000956475.1  GCA_000956505.1 | N.D.  N.D.  N.D.  N.D. | -  -  -  - | -  -  -  - | -  -  -  - |
| ZYFB012 | *Micrococcus* | P11 SW | DMS | N.D. | *Micrococcus luteus* NCTC 2665  *Micrococcus luteus* | GCA_000023205.1  GCA_000877795.1 | N.D.  N.D. | -  - | -  - | -  - |
| LZB054  ZYFD013 | *Mycobacterium*  *Mycobacterium* | ME3 SW  P11 BW | N.D.  N.D. | *dmdA A/2*  *dmdA E/2* | *Mycobacterium smegmatis* str. MC2 155  *Mycobacterium abscessus* ATCC 19977 | GCA_000015005.1  GCA_000069185.1 | N.D.  N.D. | -  - | -  - | -  - |
| LZB046  ZYFD039 | *Nocardioides*  *Nocardioides* | ME3 SW  P11 BW | N.D.  N.D. | N.D.  N.D. | *Nocardioides halotolerans* DSM 19273  *Nocardioides insulae* DSM 17944 | GCA_000422805.1  GCA_000422825.1 | N.D.  DmdA | -  WP_028660116 | -  7e-94 | -  43.09% |
| ZYFB036 | *Oceanicaulis* | P11 SW | DMS | N.D. | *Oceanicaulis alexandrii* DSM 11625  *Oceanicaulis* sp. HTCC2633 | GCA_000420265.1  GCA_000152745.1 | N.D.  N.D. | -  - | -  - | -  - |
| LZB062  LZD010  LZD026 | *Oceanicola*  *Oceanicola*  *Oceanicola* | ME3 SW  ME3 BW  ME3 BW | N.D.  DMS  DMS and MeSH | *dmdA A/2*  *dddL*  *dddL* | *Oceanicola nanhaiensis* DSM 18065  *Oceanicola batsensis* HTCC2597  *Oceanicola granulosus* HTCC2516 | GCA_000688295.1  GCA_000152725.1  GCA_000153305.1 | DddL  DddL  DmdA  DddP | WP_028285536  EAQ04071  EAR53105  EAR50963 | 4e-119 to 2e-65  5e-124 to 7e-61  4e-92 to 2e-84  0 to 7e-101 | 49.30%–73.99%  46.48%–78.28%  40.87%–43.90%  40.46%–74.94% |
| LZB009  ZYFD006 | *Paracoccus*  *Paracoccus* | ME3 SW  P11 BW | DMS  DMS | N.D.  N.D. | *Paracoccus denitrificans* PD1222  *Paracoccus aminophilus* JCM 7686  *Paracoccus yeei* ATCC BAA-599 | GCA_000203895.1  GCA_000444995.1  GCA_000622145.1 | N.D.  DddP  DddP | -  WP_020952779  WP_028718905 | -  6e-98 to 6e-92  0 to 6e-102 | -  40.00%–42.27%  43.15%–75.58% |
| LZD001  ZYFB032 | *Pelagibaca*  *Pelagibaca* | ME3 BW  P11 SW | N.D.  N.D. | N.D.  N.D. | *Pelagibaca bermudensis* HTCC2601 | GCA_000153725.1 | N.D. | - | - | - |
| LZB055 | *Phycicoccus* | ME3 SW | DMS | N.D. | *Phycicoccus jejuensis* | GCA_000720925.1 | N.D. | - | - | - |
| LZB004  ZYFD008 | *Psychrobacter*  *Psychrobacter* | ME3 SW  P11 BW | N.D.  N.D. | N.D.  N.D. | *Psychrobacter arcticus* 273-4  *Psychrobacter lutiphocae* DSM 21542  *Psychrobacter phenylpyruvicus* DSM 7000 | GCA_000012305.1  GCA_000382145.1  GCA_000685805.1 | N.D.  N.D.  N.D. | -  -  - | -  -  - | -  -  - |
| ZYFD034  LZB027 | *Rhodococcus*  *Rhodococcus* | P11 BW  ME3 SW | N.D.  N.D. | N.D.  N.D. | *Rhodococcus fascians* A44A  *Rhodococcus erythropolis* PR4 | GCA_000760735.1  GCA_000010105.1 | N.D.  N.D. | -  - | -  - | -  - |
| ZYFB035 | *Ruegeria* | P11 SW | DMS and MeSH | *dddP* | *Ruegeria pomeroyi* DSS-3  *Ruegeria lacuscaerulensis* ITI-1157  *Ruegeria mobilis* F1926  *Ruegeria mobilis* | GCA_000011965.2  GCA_000161775.1  GCA_000376545.1  GCA_000967745.1 | DmdA1  DmdA2  DddD  DddP  DddQ  DddW  DmdA  DddP  DddQ  DmdA  DddP  DmdA  DddP | WP_011047385  WP_011047644  WP_011047438  WP_044029245  WP_011047333  WP_011046214  WP_005983312  WP_005982391  WP_005978225  ENZ93420  ENZ89089  KJZ25257  KJZ21592 | 8e-89 to 2e-84  0 to 9e-108  0  0 to 2e-100  1e-144 to 2e-52  3e-106  0 to 3e-119  0 to 1e-100  7e-138 to 2e-34  0 to 7e-113  0 to 4e-102  0 to 7e-113  0 to 4e-102 | 40.53%–41.79%  41.44%–100.00%  40.74%–48.73%  40.93%–100.00%  42.05%–100.00%  100.00%  44.89%–74.32%  40.67%–91.09%  40.24%–99.48%  44.13%–76.27%  40.87%–88.78%  44.13%–76.27%  40.87%–88.78% |
| LZB024 | *Streptomyces* | ME3 SW | N.D. | N.D. | *Streptomyces anulatus*  *Streptomyces coelicolor* A3(2) | GCF_000717105.1  GCF_000203835.1 | N.D.  N.D. | - | - | - |
| LZD014  LZD018 | *Sulfitobacter*  *Sulfitobacter* | ME3 BW  ME3 BW | DMS and MeSH  DMS and MeSH | *dddL*, *dmdA A/2*  *dddP* | *Sulfitobacter pontiacus* 3SOLIMAR09  *Sulfitobacter* sp. EE-36  *Sulfitobacter mediterraneu*s KCTC 32188  *Sulfitobacter donghicola* DSW-25 | GCA_000647675.1  GCA_000152605.1  GCA_000622345.1  GCA_000622405.1 | DmdA  DddL  DmdA  DddL  DmdA1  DmdA2  DmdA3  DddP  DddL  DddP | KAJ31754  KAJ31922  EAP83955  EAP83768  KIN79610  KIN79163  KIN76909  KIN77078  KIN66932  KIN66723 | 2e-88 to 3e-82  8e-166 to 5e-62  7e-92 to 3e-81  8e-166 to 5e-62  2e-96 to 5e-85  3e-103 tot 6e-98  7e-178 to 1e-111  0 to 1e-103  2e-133 to 1e-64  0 to 9e-105 | 40.64%–42.02%  47.20%–100.00%  41.44%–42.30%  47.20%–100.00%  40.58%–42.90%  40.27%–43.45%  45.18%–70.60%  42.16%–84.44%  48.83%–80.54%  41.60%–82.10% |

N.D., not detected of any DMSP dependent product (DMS or MeSH), bacterial DMSP degrading genes (*ddd* or *dmdA*), or DMSP degradation enzyme homologue in reference genomes based on the thresholds of E-value ≤ 1e-5, identity ≥ 40%, coverage ≥ 70% and length difference ≤ 20% of the protein sequences. ‘SW’ stands for surface seawater; ‘BW’ stands for bottom seawater.

**Table S3** Reference protein sequences of the ratified DMSP demethylation and cleavage enzymes

| Protein | Source | Accession Number | Reference |
| --- | --- | --- | --- |
| DmdA | *Ruegeria pomeroyi* DSS-3 | AAV95190 | Howard *et al*., 2006 |
|  | *Pelagibacter ubique* HTCC1062 | WP_011281570 | Howard *et al*., 2006 |
|  | *Dinoroseobacter shibae* DFL 12 | WP_012178987 | Howard *et al*., 2008 |
|  | *marine gammaproteobacterium* HTCC2080 | WP_007233625 | Howard *et al*., 2008 |
|  | *Candidatus* Pelagibacter sp. HTCC7211 | WP_008546106 | Howard *et al.*, 2011 |
|  | *Candidatus* Puniceispirillum marinum IMCC1322 | WP_013044947 | Howard *et al.*, 2011 |
| DddD | *Marinomonas* sp. MWYL1 | ABR72937 | Todd *et al*., 2007 |
|  | *Oceanimonas doudoroffii* | AEQ39135 | Curson *et al*., 2012 |
|  | *Psychrobacter* sp. J466 | ACY02894 | Curson *et al.*, 2010 |
|  | *Halomonas* sp. HTNK1 | ACV84065 | Todd *et al*., 2010 |
|  | *Sinorhizobium fredii* NGR234 | AAQ87407 | Todd *et al*., 2007 |
|  | *Burkholderia ambifaria* AMMD | WP_011659284 | Todd *et al*., 2007 |
|  | *Pseudomonas* sp. J465 | ACY01992 | Curson *et al.*, 2010 |
| DddL | *Sulfitobacter* sp. EE-36 | ADK55772 | Curson *et al*., 2008 |
|  | *Rhodobacter sphaeroides* 2.4.1 | YP_351475 | Curson *et al*., 2008 |
|  | *Labrenzia aggregata* LZB033 | KP639184 | Curson *et al*., 2017 |
|  | *Ahrensia marina* LZD062 | KP639183 | This study |
| DddP | *Roseovarius nubinhibens* ISM | EAP77700 | Todd *et al*., 2009 |
|  | *Ruegeria pomeroyi* DSS-3 | WP_044029245 | Todd *et al*., 2011 |
|  | *Phaeobacter inhibens* DSM 17395 | AFO91571 | Burkhardt *et al.*, 2017 |
|  | *Oceanimonas doudoroffii* DSM 7028 | AEQ39091 | Curson *et al*., 2012 |
|  | *Oceanimonas doudoroffii* DSM 7028 | AEQ39103 | Curson *et al*., 2012 |
|  | *Aspergillus oryzae* RIB40 | BAE62778 | Todd *et al*., 2009 |
|  | *Fusarium graminearum* PH-1 | XP_389272 | Todd *et al*., 2009 |
|  | *Candidatus* Puniceispirillum marinum | WP_013046297 | Choi *et al*., 2015 |
| DddQ | *Ruegeria pomeroyi* DSS-3 | WP_011047333 | Todd *et al*., 2011 |
|  | *Roseovarius nubinhibens* ISM | EAP76002 | Todd *et al*., 2011 |
|  | *Roseovarius nubinhibens* ISM | EAP76001 | Todd *et al*., 2011 |
|  | *Ruegeria lacuscaerulensis* ITI1157 | WP_005978225 | Li *et al.*, 2014 |
|  | GOS databases | ECW91654 | Todd *et al*., 2011 |
|  | GOS databases | EBP74803 | Todd *et al*., 2011 |
|  | GOS databases | ECX82089 | Todd *et al*., 2011 |
| DddW | *Ruegeria pomeroyi* DSS-3 | AAV93771 | Todd *et al*., 2012 |
| DddY | *Alcaligenes faecalis* M3A | ADT64689 | Curson *et al.*, 2011 |
|  | *Shewanella putrefaciens* CN-32 | ABP77243 | Curson *et al.*, 2011 |

**Table S4** Oligonucleotide primers used in this study.

| Primer name | Sequence (5’ to 3’) | Use | Reference |
| --- | --- | --- | --- |
| 27F | AGAGTTTGATCCTGGCTCAG | Amplification of cultivated strains 16S rRNA gene | Lane *et al*., 1991 |
| 1492R | GGTTACCTTGTTACGACTT |
|  |  |  |  |
| 515F | GTGCCAGCMGCCGCGG | Preparing partial 16S rRNA genes amplicon for pyrosequencing | Chen *et al*., 2016 |
| 907R | CCGTCAATTCMTTTRAGTTT |
|  |  |  |  |
| Eub338F | ACTCCTACGGGAGGCAGCAG | qPCR of 16S rRNA gene | Yin *et al*., 2013 |
| Eub518R | ATTACCGCGGCTGCTGG |
|  |  |  |  |
| M13F | GTAAAACGACGGCCAG | Sequencing primers for T-A clone fragments inserted in pUCm-T vector | Yin *et al*., 2013 |
| M13R | GTTTTCCCAGTCACGAC |
|  |  |  |  |
| *dddD*f | ACCAACGTCATTGCAGGACC | PCR detection of *dddD* in representative cultivated strains | Raina *et al*., 2009 |
| *dddD*r | TGTGCGTGTTCTTCCGGTG |
|  |  |  |  |
| *dddL*f | CTGGGAATACGGCTACGAGA | PCR detection of *dddL* in representative cultivated strains | Raina *et al*., 2009 |
| *dddL*r | GTTCAAGATCAGCGATCCGG |
|  |  |  |  |
| DddPUf | ATGTTCGACCCGATGAACathmgntaygc | PCR detection of *dddP* in representative cultivated strains | This study |
| DddPUr | CCGCACTCCTGGAACcanggrttngt |
|  |  |  |  |
| dmdAUF160 | GTICARITITGGGAYGT | PCR detection of *dmdA* in representative cultivated strains | Varaljay et al., 2010 |
| dmdAUR697 | TCIATICKITCIATIAIRTTDGG |
|  |  |  |  |
| A/1-spFP | ATGGTGATTTGCTTCAGTTTCT | PCR detection of *dmdA* in representative cultivated strains and qPCR | Varaljay et al., 2010 |
| A/1-spRP | CCCTGCTTTGACCAACC |
|  |  |  |  |
| A/2-spFP | CGATGAACATTGGTGGGTTTCTA | PCR detection of *dmdA* in representative cultivated strains and qPCR | Varaljay et al., 2010 |
| A/2-spRP | GCCATTAGGTCGTCTGATTTTGG |
|  |  |  |  |
| B/3-spFP | GATGTCTCCTGCCAACGTCAGGTCGA | PCR detection of *dmdA* in representative cultivated strains and qPCR | Varaljay et al., 2010 |
| B/3-spRP | ACCGGGTCATTGATCATGCCTGCG |
|  |  |  |  |
| C/2-spFP | AGATGAAAATGCTGGAATGATA  AATG | qPCR | Varaljay et al., 2010 |
| C/2-spRP | AAATCTTCAGACTTTGGACCTTG |
|  |  |  |  |
| D/1-spFP | AGATGTTATTATTGTCCAATAATT  GATG | qPCR | Varaljay et al., 2010 |
| D/1-spRP | ATCCACCATCTATCTTCAGCTA |
|  |  |  |  |
| D/3-spFP | AATGGTGGATTTCTATTGCAG  ATAC | qPCR | Varaljay et al., 2010 |
| D/3-spRP | GATTTTGGACCTTGTACAGCCA |
|  |  |  |  |
| E/2-spFP | CATGTTCAGATCTGGGACGT | PCR detection of *dmdA* in representative cultivated strains and qPCR | Varaljay et al., 2010 |
| E/2-spRP | AGCGGCACATACATGCACT |
|  |  |  |  |
| dddP_874F | AAYGAAATWGTTGCCTTTGA | qPCR | Levine *et al*., 2012 |
| dddP_971R | GCATDGCRTAAATCATATC |
|  |  |  |  |
| *dddP*F | GCAGCTCTGGAACGCCCATA | Clone library construction | Peng *et al*., 2012 |
| *dddP*R | GCATCAGGCAGCCGTATTTC |

**Table S5** Data of 16S rRNA gene pyrosequencing and richness and diversity estimators.

| Station | Sampling layer | Sequence numbers with good quality | Bacterial sequence number after rarefying | OTU numbers | Shannon index | Simpson index | Good’s coverage | Chao 1’ |
| --- | --- | --- | --- | --- | --- | --- | --- | --- |
| P03 | SW | 31017 | 17774 | 549 | 6.56 | 0.0279 | 0.99 | 729.76 |
|  | BW | 30626 | 17774 | 1522 | 7.86 | 0.0157 | 0.97 | 2141.62 |
| P05 | SW | 31667 | 17774 | 894 | 6.61 | 0.0417 | 0.98 | 1419.23 |
|  | BW | 27010 | 17774 | 1020 | 6.40 | 0.04656 | 0.97 | 1599.96 |
| P07 | SW | 37122 | 17774 | 735 | 6.23 | 0.0567 | 0.99 | 1037.16 |
|  | BW | 25367 | 17774 | 1685 | 7.96 | 0.0302 | 0.96 | 2390.88 |
| P10 | SW | 43104 | 17774 | 661 | 6.17 | 0.0591 | 0.99 | 824.26 |
|  | BW | 28770 | 17774 | 1076 | 7.48 | 0.0199 | 0.98 | 1425.20 |
| P12 | SW | 37062 | 17774 | 604 | 5.81 | 0.0711 | 0.99 | 789.05 |
|  | 100 m | 42718 | 17774 | 662 | 6.50 | 0.0343 | 0.99 | 855.12 |
|  | BW | 28191 | 17774 | 709 | 6.88 | 0.0206 | 0.99 | 856.35 |

‘SW’ stands for surface seawater; ‘BW’ stands for bottom seawater.

**Table S6** Summary of number of effective *dddP* clones classified into different OTUs, richness and diversity estimators and coverage values.

| Station | Sampling layer | Effective clones | OTU1 | OTU2 | OTU3 | OTU4 | OTU5 | OTU6 | OTU7 | OTU8 | OTU9 | OTU10 | OTU11 | OTU12 | OTU13 | chao’ 1 | Shannon index | Simpson index | Good’s coverage |
| --- | --- | --- | --- | --- | --- | --- | --- | --- | --- | --- | --- | --- | --- | --- | --- | --- | --- | --- | --- |
| P03 | SW | 32 | 10 | 16 | 5 | 0 | 0 | 0 | 0 | 1 | 0 | 0 | 0 | 0 | 0 | 4 | 1.11 | 0.35 | 0.97 |
|  | BW | 37 | 3 | 5 | 23 | 3 | 0 | 0 | 1 | 1 | 0 | 0 | 0 | 1 | 0 | 10 | 1.27 | 0.4 | 0.92 |
| P05 | SW | 25 | 4 | 12 | 5 | 0 | 0 | 2 | 2 | 0 | 0 | 0 | 0 | 0 | 0 | 4 | 0.9 | 0.5 | 0.97 |
|  | BW | 25 | 7 | 14 | 2 | 0 | 0 | 0 | 0 | 0 | 0 | 2 | 0 | 0 | 0 | 4 | 1.09 | 0.38 | 1 |
| P10 | SW | 26 | 4 | 10 | 6 | 3 | 0 | 2 | 0 | 0 | 0 | 0 | 0 | 0 | 1 | 5 | 1.37 | 0.28 | 1 |
|  | BW | 24 | 11 | 8 | 5 | 0 | 0 | 0 | 0 | 0 | 0 | 0 | 0 | 0 | 0 | 3 | 1.05 | 0.34 | 1 |
| P11 | SW | 29 | 24 | 1 | 3 | 0 | 1 | 0 | 0 | 0 | 0 | 0 | 0 | 0 | 0 | 6 | 1.57 | 0.22 | 0.96 |
|  | BW | 27 | 9 | 0 | 15 | 1 | 0 | 0 | 0 | 0 | 1 | 0 | 1 | 0 | 0 | 8 | 1.06 | 0.4 | 0.89 |
| P12 | SW | 29 | 9 | 14 | 6 | 0 | 0 | 0 | 0 | 0 | 0 | 0 | 0 | 0 | 0 | 5 | 0.62 | 0.69 | 0.93 |
|  | 100 m | 23 | 2 | 19 | 2 | 0 | 0 | 0 | 0 | 0 | 0 | 0 | 0 | 0 | 0 | 3 | 0.58 | 0.68 | 1 |
| ME3 | SW | 37 | 25 | 0 | 8 | 0 | 3 | 0 | 0 | 0 | 1 | 0 | 0 | 0 | 0 | 3 | 1.04 | 0.35 | 1 |

‘SW’ stands for surface seawater; ‘BW’ stands for bottom seawater.

**Table S7** The copy numbers of DMSP degrading genes and 16S rRNA gene (copies L^-1^) in the ECS.

| Station | Sampling layer | *dmdA* A/1 | *dmdA* A/2 | *dmdA* B/3 | *dmdA* C/2 | *dmdA* D/1 | *dmdA* D/3 | *dmdA* E/2 | *dddP* | 16S rDNA |
| --- | --- | --- | --- | --- | --- | --- | --- | --- | --- | --- |
| P03 | SW | 1.73E+07 | 2.43E+07 | 2.03E+07 | 1.66E+06 | 2.91E+08 | 9.05E+07 | 1.71E+08 | 1.66E+08 | 5.62E+09 |
|  | BW | 4.80E+06 | 2.02E+07 | 5.24E+07 | 6.89E+05 | 7.78E+07 | 7.86E+07 | 3.19E+07 | 2.60E+07 | 2.21E+09 |
| P05 | SW | 2.01E+07 | 3.62E+07 | 3.15E+07 | 1.10E+07 | 4.41E+08 | 1.46E+08 | 3.24E+07 | 8.42E+07 | 3.40E+09 |
|  | BW | 2.03E+07 | 4.59E+07 | 7.13E+07 | 2.03E+06 | 2.99E+08 | 1.79E+08 | 3.44E+07 | 5.54E+07 | 2.68E+09 |
| P07 | SW | 2.44E+07 | 4.20E+07 | 5.02E+07 | 1.53E+07 | 3.09E+08 | 2.29E+08 | 2.02E+07 | 7.05E+07 | 3.13E+09 |
|  | BW | 6.15E+06 | 2.62E+07 | 5.46E+07 | 1.48E+06 | 1.55E+08 | 1.12E+08 | 1.01E+07 | 2.02E+07 | 1.36E+09 |
| P10 | SW | 6.16E+07 | 6.92E+07 | 9.14E+07 | 4.30E+07 | 6.56E+08 | 4.68E+08 | 7.38E+07 | 2.34E+08 | 1.07E+10 |
|  | BW | 2.53E+06 | 9.29E+06 | 1.55E+07 | 1.72E+06 | 6.04E+07 | 6.09E+07 | 2.81E+06 | 6.04E+06 | 1.11E+09 |
| P12 | SW | 1.74E+06 | 3.99E+05 | 1.33E+06 | 3.70E+06 | 4.92E+06 | 7.42E+06 | 2.71E+05 | 8.53E+05 | 6.61E+07 |
|  | 100 m | 8.82E+05 | 8.38E+04 | 7.44E+05 | 3.86E+06 | 2.51E+06 | 1.69E+06 | 1.71E+05 | 1.38E+05 | 4.75E+07 |
|  | BW | 8.60E+03 | 6.25E+03 | 3.84E+03 | 8.01E+04 | 5.14E+04 | 5.00E+04 | 2.58E+04 | 1.91E+04 | 7.81E+06 |

‘SW’ stands for surface seawater; ‘BW’ stands for bottom seawater.

**Table** **S8** Growth of the representative isolates which can grow with DMSP as the sole carbon source or with mixed carbon source*

| Bacterial strains | DMSP | Mixed carbon source |
| --- | --- | --- |
| ***Alphaproteobacteria*** |  |  |
| *Sulfitobacter* sp. LZD018 | + | + |
| *Ahrensia* sp. LZD051 | w | + |
| *Ahrensia* sp. LZD062 | + | + |
| ***Gammaproteobacteria*** |  |  |
| *Halomonas* sp. LZD012 | + | + |

*Only the strains which are able to grow on DMSP as sole carbon source are listed; for total 57 representative information, see Table S3.

+, Positive growth; w, weak growth. Growth was considered positive (*P* < 0.01) or weak (*P* < 0.05) where the averages of maximal absorbance of triplicate wells were significantly greater (Student’s t-test) than those of no organic carbon source controls.

**Table S9** DMSP consumption of dmdA-like gene containing but non-detectable MeSH producing strains.

|  | MeSH production (Peak Area) | | | DMS production (nmol in 300 ul culture) | | | Remained DMSP (nmol in 300 ul culture) | | | Student’s t-test^#^ |
| --- | --- | --- | --- | --- | --- | --- | --- | --- | --- | --- |
|  | Replicate 1 | Replicate 2 | Replicate 3 | Replicate 1 | Replicate 2 | Replicate 3 | Replicate 1 | Replicate 2 | Replicate 3 |  |
| *Mycobacterium* sp. LZD054 | N.D. | N.D. | N.D. | N.D. | N.D. | N.D. | 483.64 | 480.50 | 319.43 | P > 0.05 |
| *Henriciella* sp. ZYFB017 | N.D. | N.D. | N.D. | N.D. | N.D. | N.D. | 452.53 | 538.77 | 597.57 | P > 0.05 |
| *Aurantimonas* sp. ZYFD019 | N.D. | N.D. | N.D. | 3.06 | 3.57 | 3.27 | 549.65 | 549.35 | 588.27 | P > 0.05 |
| *Mycobacterium* sp. ZYFD013 | N.D. | N.D. | N.D. | N.D. | N.D. | N.D. | 636.52 | 574.97 | N.A. | P > 0.05 |
| *R*. *pomeroyi* DSS-3 | 3211 | 4376 | 1516 | 6.20 | 7.00 | 4.87 | 371.34 | 400.59 | 404.22 | P > 0.05 |
| *R*. *nubinhibens* ISM | 29504 | 27534 | 28910 | 8.21 | 7.87 | 8.29 | 2.79 | 2.91 | N.A. | P < 0.05 |
| Media control | N.D. | N.D. | N.D. | N.D. | N.D. | N.D. | N.A. | 645.34 | 461.89 |  |

N.D. stands for MeSH or DMS production from DMSP was not detectable; N.A. stands for not available;

#, student’s t-test was conducted to test the significance of the difference between the remaining DMSP in the culture and in the media control. Since these strains cannot grow with DMSP as sole carbon source, final concentration of 2 mM DMSP was added with the mixed carbon source into the media.

**Reference for supplementary materials**

Balch, W. E., Fox, G. E., Magrum, L. J., Woese, C. R., and Wolfe, R. S. (1979). Methanogens: reevaluation of a unique biological group. *Microbiol. Rev.* 43, 260–96. Available at: http://www.ncbi.nlm.nih.gov/pubmed/390357 [Accessed April 14, 2018].

Burkhardt, I., Lauterbach, L., Brock, N. L., and Dickschat, J. S. (2017). Chemical differentiation of three DMSP lyases from the marine Roseobacter group. *Org. Biomol. Chem.* 15, 4432–4439. doi:10.1039/C7OB00913E.

Chen, H., Zhang, H., Xiong, J., Wang, K., Zhu, J., Zhu, X., et al. (2016). Successional trajectories of bacterioplankton community over the complete cycle of a sudden phytoplankton bloom in the Xiangshan Bay, East China Sea. *Environ. Pollut.* 219, 750–759. doi:10.1016/J.ENVPOL.2016.07.035.

Choi, D. H., Park, K.-T., An, S. M., Lee, K., Cho, J.-C., Lee, J.-H., et al. (2015). Pyrosequencing Revealed SAR116 Clade as Dominant dddP-Containing Bacteria in Oligotrophic NW Pacific Ocean. *PLoS One* 10, e0116271. doi:10.1371/journal.pone.0116271.

Curson, A. R. J., Fowler, E. K., Dickens, S., Johnston, A. W. B., and Todd, J. D. (2012). Multiple DMSP lyases in the γ-proteobacterium Oceanimonas doudoroffii. *Biogeochemistry* 110, 109–119. doi:10.1007/s10533-011-9663-2.

Curson, A. R. J., Liu, J., Bermejo Martínez, A., Green, R. T., Chan, Y., Carrión, O., et al. (2017). Dimethylsulfoniopropionate biosynthesis in marine bacteria and identification of the key gene in this process. *Nat. Microbiol.* 2, 17009. doi:10.1038/nmicrobiol.2017.9.

Curson, A. R. J., Rogers, R., Todd, J. D., Brearley, C. A., and Johnston, A. W. B. (2008). Molecular genetic analysis of a dimethylsulfoniopropionate lyase that liberates the climate-changing gas dimethylsulfide in several marine α-proteobacteria and Rhodobacter sphaeroides. *Environ. Microbiol.* 10, 757–767. doi:10.1111/j.1462-2920.2007.01499.x.

Curson, A. R. J., Sullivan, M. J., Todd, J. D., and Johnston, A. W. B. (2010). Identification of genes for dimethyl sulfide production in bacteria in the gut of Atlantic Herring (Clupea harengus). *ISME J.* 4, 144–146. doi:10.1038/ismej.2009.93.

Curson, A. R. J., Sullivan, M. J., Todd, J. D., and Johnston, A. W. B. (2011). DddY, a periplasmic dimethylsulfoniopropionate lyase found in taxonomically diverse species of Proteobacteria. *ISME J.* 5, 1191–1200. doi:10.1038/ismej.2010.203.

Howard, E. C., Henriksen, J. R., Buchan, A., Reisch, C. R., Bürgmann, H., Welsh, R., et al. (2006). Bacterial taxa that limit sulfur flux from the ocean. *Science* 314, 649–652. doi:10.1126/science.1130657.

Howard, E. C., Sun, S., Biers, E. J., and Moran, M. A. (2008). Abundant and diverse bacteria involved in DMSP degradation in marine surface waters. *Environ. Microbiol.* 10, 2397–2410. doi:10.1111/j.1462-2920.2008.01665.x.

Howard, E. C., Sun, S., Reisch, C. R., del Valle, D. A., Bürgmann, H., Kiene, R. P., et al. (2011). Changes in dimethylsulfoniopropionate demethylase gene assemblages in response to an induced phytoplankton bloom. *Appl. Environ. Microbiol.* 77, 524–31. doi:10.1128/AEM.01457-10.

Lane, D. J. (1991). 16S/23S rRNA sequencing. *Nucleic acid Tech. Bact. Syst.* Available at: https://ci.nii.ac.jp/naid/10005795102/ [Accessed April 15, 2018].

Levine, N. M., Varaljay, V. A., Toole, D. A., Dacey, J. W. H., Doney, S. C., and Moran, M. A. (2012). Environmental, biochemical and genetic drivers of DMSP degradation and DMS production in the Sargasso Sea. *Environ. Microbiol.* 14, 1210–1223. doi:10.1111/j.1462-2920.2012.02700.x.

Li, C.-Y., Wei, T.-D., Zhang, S.-H., Chen, X.-L., Gao, X., Wang, P., et al. (2014). Molecular insight into bacterial cleavage of oceanic dimethylsulfoniopropionate into dimethyl sulfide. *Proc. Natl. Acad. Sci. U. S. A.* 111, 1026–31. doi:10.1073/pnas.1312354111.

Peng, M., Xie, Q., Hu, H., Hong, K., Todd, J. D., Johnston, A. W. B., et al. (2012). Phylogenetic diversity of the *dddP* gene for dimethylsulfoniopropionate-dependent dimethyl sulfide synthesis in mangrove soils. *Can. J. Microbiol.* doi:10.1139/w2012-019.

Raina, J.-B., Tapiolas, D., Willis, B. L., and Bourne, D. G. (2009). Coral-associated bacteria and their role in the biogeochemical cycling of sulfur. *Appl. Environ. Microbiol.* 75, 3492–3501. doi:10.1128/AEM.02567-08.

Steinsbu, B. O., Thorseth, I. H., Nakagawa, S., Inagaki, F., Lever, M. A., Engelen, B., et al. (2010). Archaeoglobus sulfaticallidus sp. nov., a thermophilic and facultatively lithoautotrophic sulfate-reducer isolated from black rust exposed to hot ridge flank crustal fluids. *Int. J. Syst. Evol. Microbiol.* 60, 2745–2752. doi:10.1099/ijs.0.016105-0.

Todd, J. D., Curson, A. R. J., Dupont, C. L., Nicholson, P., and Johnston, A. W. B. (2009). The *dddP* gene, encoding a novel enzyme that converts dimethylsulfoniopropionate into dimethyl sulfide, is widespread in ocean metagenomes and marine bacteria and also occurs in some Ascomycete fungi. *Environ. Microbiol.* 11, 1376–1385. doi:10.1111/j.1462-2920.2009.01864.x.

Todd, J. D., Curson, A. R. J., Kirkwood, M., Sullivan, M. J., Green, R. T., and Johnston, A. W. B. (2011). DddQ, a novel, cupin-containing, dimethylsulfoniopropionate lyase in marine roseobacters and in uncultured marine bacteria. *Environ. Microbiol.* 13, 427–438. doi:10.1111/j.1462-2920.2010.02348.x.

Todd, J. D., Curson, A. R. J., Nikolaidou-Katsaraidou, N., Brearley, C. A., Watmough, N. J., Chan, Y., et al. (2010). Molecular dissection of bacterial acrylate catabolism - unexpected links with dimethylsulfoniopropionate catabolism and dimethyl sulfide production. *Environ. Microbiol.* 12, 327–343. doi:10.1111/j.1462-2920.2009.02071.x.

Todd, J. D., Kirkwood, M., Newton-Payne, S., and Johnston, A. W. B. (2012). DddW, a third DMSP lyase in a model Roseobacter marine bacterium, Ruegeria pomeroyi DSS-3. *ISME J.* 6, 223–226. doi:10.1038/ismej.2011.79.

Todd, J. D., Rogers, R., Li, Y. G., Wexler, M., Bond, P. L., Sun, L., et al. (2007). Structural and regulatory genes required to make the gas dimethyl sulfide in bacteria. *Science* 315, 666–9. doi:10.1126/science.1135370.

Varaljay, V. A., Howard, E. C., Sun, S., and Moran, M. A. (2010). Deep sequencing of a dimethylsulfoniopropionate-degrading gene (dmdA) by using PCR primer pairs designed on the basis of marine metagenomic data. *Appl. Environ. Microbiol.* 76, 609–617. doi:10.1128/AEM.01258-09.

Yin, Q., Fu, B., Li, B., Shi, X., Inagaki, F., and Zhang, X.-H. (2013). Spatial Variations in Microbial Community Composition in Surface Seawater from the Ultra-Oligotrophic Center to Rim of the South Pacific Gyre. *PLoS One* 8, e55148. doi:10.1371/journal.pone.0055148.
